# RASAL2 regulates the cell cycle and cyclin D1 expression through PI3K/AKT signalling in prostate tumorigenesis

**DOI:** 10.1038/s41420-022-01069-3

**Published:** 2022-06-06

**Authors:** Qi Wang, Shiqi Wu, Yanan Gu, Hua Liang, Fei He, Xinyang Wang, Dalin He, Kaijie Wu

**Affiliations:** 1 Department of Urology, First Affiliated Hospital of Xi’an Jiaotong University, Xi’an, 710061 P.R. China; 2 Department of Pathology, First Affiliated Hospital of Xi’an Jiaotong University, Xi’an, 710061 P.R. China

**Keywords:** Prostate cancer, Oncogenes

## Abstract

Prostate cancer (PCa) growth and progression are uniquely dependent on androgens, making the androgen receptor pathway a prime target for therapy; however, cancer progression to androgen independence leads to treatment failure and poor prognosis. In recent years, alternative therapeutic pathways for PCa have been extensively explored, such as the PTEN/PI3K/AKT pathway, cell cycle, and DNA repair. In the present study, we discovered that RASAL2, a RAS-GTPase-activating protein, acted as an oncogene to regulate cancer cell proliferation and the cell cycle and contributed to tumorigenesis via the PI3K/AKT/cyclin D1 pathway. First, RASAL2 expression was higher in PCa tumour and metastatic lymph node tissues than in matched adjacent nontumor tissues and was associated with higher PCa tumour stage, Gleason score and poorer prognosis. Mechanistically, we found that RASAL2 promoted tumour cell proliferation, the transition from G1 to S phase in vitro and tumour growth in vivo. Furthermore, we demonstrated that RASAL2 facilitated phosphorylation of AKT, which in turn increased the expression of cyclin D1 encoded by the CCND1 gene. In addition, there was a positive correlation between the expression of RASAL2 and cyclin D1 in subcutaneous xenografts and clinical specimens. Taken together, these findings indicate that RASAL2 plays an oncogenic role in prostate cancer and may promote PCa tumorigenesis through PI3K/AKT signalling and cyclin D1 expression.

## Introduction

Prostate cancer (PCa) has the highest cancer incidence and second highest mortality in men in the United States, and it is estimated that there may be 248,530 new cases in 2021, causing total mortality of more than 30,000 [1]. Inhibition of androgen receptor (AR) signalling is the primary method of treatment for metastatic PCa, and androgen deprivation therapy (ADT) in combination with anti-androgen drugs or docetaxel chemotherapy significantly prolongs life expectancy in PCa patients. Second-generation AR inhibitors, such as enzalutamide, have even improved survival rates in patients with metastatic castration-resistant prostate cancer (mCRPC). However, acquired drug resistance leads to poor patient prognosis [2]. Therefore, targeting alternative pathways to prevent and treat refractory PCa is urgent.

Alterations involving PTEN loss and PI3K/AKT pathway genes are common in PCa, and there may be crosstalk between PI3K/AKT and AR signalling, suggesting opportunities for combination therapy. The cell cycle is the governing mechanism of cell division, and disruption of this pathway may lead to uncontrolled cell proliferation. Genomic alterations associated with cell cycle regulation are present in up to 25% of patients with mCRPC [3, 4]. Genomic alterations in the cell cycle and the PI3K/AKT pathway have been identified as potential targets for alternative treatment of mCRPC patients resistant to second-generation anti-androgen therapies, and several targeted agents have entered clinical trials [2]. Therefore, exploring new molecules that can specifically regulate these pathways may represent a promising strategy for targeting PCa.

RAS-GTPase-activating proteins (GAPs) are key regulators of RAS proteins that turn off RAS by catalysing RAS-mediated GTP hydrolysis [5]. As a unique member of the RAS-GAP family, RASAL2 has been found to have inconsistent roles across different tumours in previous studies [6]. RASAL2 is a reported tumour suppressor in luminal B breast cancer, ovarian cancer, pancreatic ductal carcinoma, nasopharyngeal carcinoma and malignant astrocytoma [6, 7]. In contrast, RASAL2 is upregulated in triple-negative breast cancer and oestrogen-receptor negative breast cancer and is associated with poor prognosis, early metastasis, and tumour recurrence [8–10]. Interestingly, a controversial role for RASAL2 has emerged in colorectal cancer, lung cancer and liver cancer [6, 11, 12]. In our previous study, we reported that RASAL2 was downregulated in bladder and kidney cancers, which regulated angiogenesis [13, 14], while RASAL2 also affected stemness and epithelial-mesenchymal transition in bladder cancer [15]. However, the role of RASAL2 in PCa progression is not clear.

In this study, for the first time, we demonstrated that RASAL2 was upregulated in prostate cancer and associated with the cell cycle, tumour growth and poor disease prognosis, in which the PI3K/AKT signalling pathway and cyclin D1 expression played an important role. Therefore, we suggest that RASAL2 may represent a new potential target for PCa treatment.

## Results

### RASAL2 is upregulated in human PCa tissues and is positively correlated with tumour progression

**Fig. 1:**
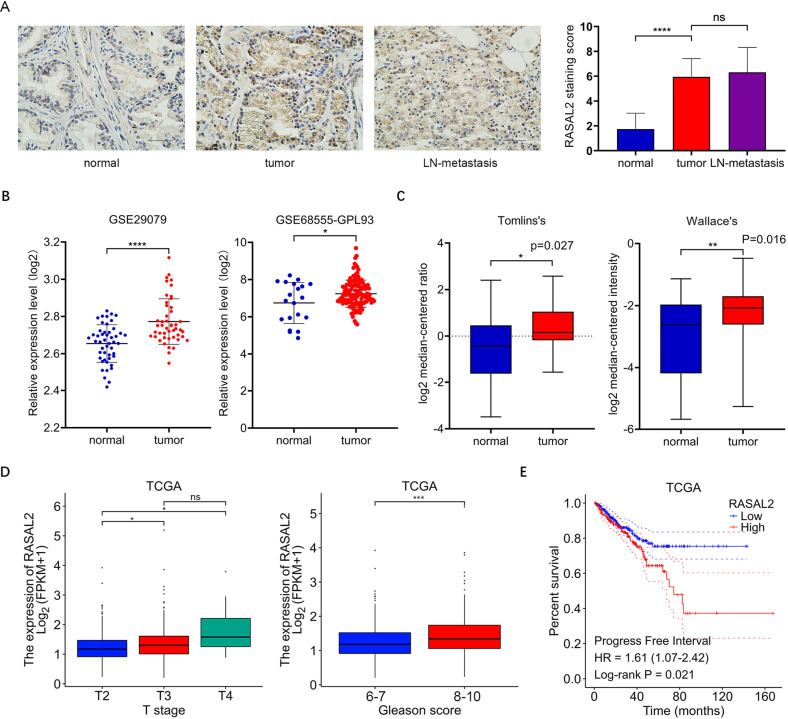
Expression of RASAL2 in PCa and normal prostate tissues. **A** Immunohistochemical staining of RASAL2 in prostate cancer, adjacent nontumor tissue and metastatic lymph nodes (*n* = 19). The scale bar is 100 μm. **B** RASAL2 mRNA expression in normal prostate tissues and PCa tissues from the GEO database (GSE29079 and GSE68555). **C** RASAL2 mRNA expression in normal prostate tissues and PCa tissues from Oncomine databases (Tomlin’s and Wallace’s). **D** Clinical correlation of RASAL2 with PCa T-stage and Gleason score from TCGA database. **E** Prognostic data for high and low expression of RASAL2 derived from the TCGA database.

To clarify the expression characteristics of RASAL2 in PCa, we performed immunohistochemistry on 19 matched groups of PCa tumour and metastatic lymph node tissues compared to their adjacent nontumor tissues (Table 1). The results showed that RASAL2 protein expression levels were higher in PCa tumour and metastatic lymph node tissues than in nontumor tissues. (Fig. 1A and Supplemental Fig. 1A). Similarly, RASAL2 mRNA levels were elevated in tumour tissues in datasets from the GEO and Oncomine databases (Fig. 1B, C). To further understand the clinical relevance of the differential expression of RASAL2, we analyzed a TCGA PCa cohort, which revealed that expression levels of RASAL2 were positively correlated with both T stage and Gleason score (Fig. 1D). Moreover, Kaplan–Meier curves showed that patients with higher RASAL2 expression had poorer progression-free survival (Fig. 1E) and overall survival (Supplemental Fig. 1B). Collectively, RASAL2 is upregulated in PCa tissues and is associated with aggressive disease and poor patient prognosis.

**Table 1:** Clinical features of prostate cancer patient specimens.

Age at diagnosis (years)	53~77
Range (mean ± SD)	66.6 ± 7
Preoperative PSA (nmol/L)	
<4	2
4–10	1
>10	16
Gleason score	
7	4
8	3
9	9
10	1
Unknown	2
pTs tage	
T2a	1
T2c	1
T3a	1
T3b	8
T4	8

*N* = 19

### RASAL2 promotes PCa cell proliferation and alters the cell cycle in vitro

**Fig. 2:**
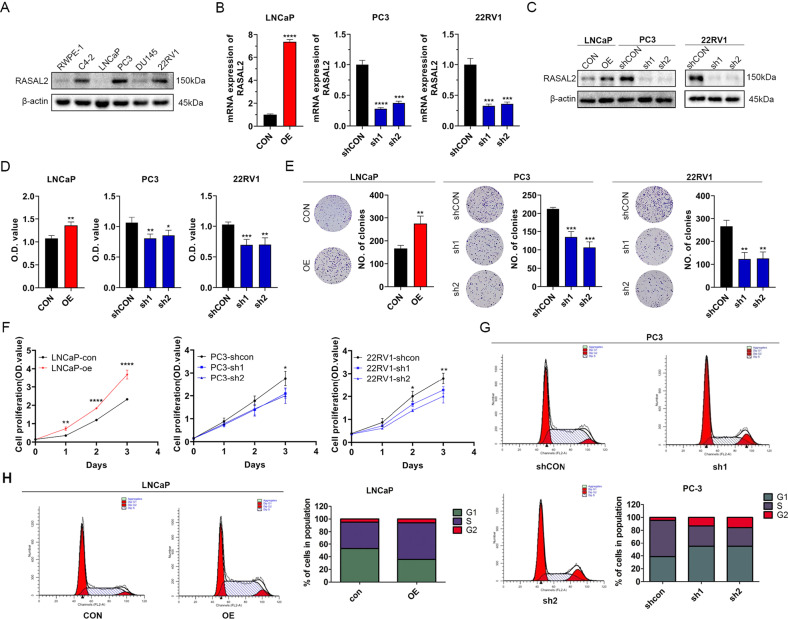
Expression of RASAL2 in PCa cell lines and the effects of RASAL2 on the proliferation of PCa cell lines. **A** Western blotting analysis of RASAL2 expression levels in human nontumor prostate and PCa cell lines. **B**, **C** Quantitative real-time RT–PCR and Western blotting analysis of RASAL2 expression in 22RV1 or PC3 cell lines transfected with RASAL2 shRNAs and shControl and LNCaP cell lines infected with RASAL2 lentivirus and negative control. **D** Cell activity assay for established cell lines. **E** Clone formation experiments with quantitative results on established cell lines. **F** Cell proliferation experiments on established cell lines. **G**, **H** Cell cycle analysis of PC3 and LNCaP cell lines using flow cytometry.

Through Western blot experiments in different PCa cell lines, we observed increased expression levels of RASAL2 in PC3, 22RV1 and C4-2 cell lines than in LNCaP and DU145 cell lines (Fig. 2A). To investigate the effect of RASAL2 on the function and pathway of PCa, we generated stable knockdown (PC3 and 22RV1) and overexpression (LNCaP) cell lines (Fig. 2B, C). First, we assessed cell viability in these established cell lines. The viability of PCa cells increased after RASAL2 overexpression and decreased after RASAL2 knockdown (Fig. 2D). Based on these preliminary data, we performed additional experiments associated with cell proliferation, such as colony formation and CCK8 assays (Fig. 2E, F). RASAL2 overexpression in LNCaP cells facilitated cell proliferation and colony formation, while RASAL2 knockdown in PC3 and 22RV1 cells inhibited proliferation. Cell cycle distribution is directly linked with cell proliferation; therefore, we supposed that RASAL2 regulated proliferation by affecting the PCa cell cycle, and flow cytometry was performed to assess the cell cycle distribution of LNCaP and PC3 sublines. As expected, the proportion of cells entering the S phase in the RASAL2-overexpressing cell line was substantially higher than that in the control group, whereas fewer RASAL2-knockdown cells entered S phase (Fig. 2G, H). These results suggest that high expression of RASAL2 in PCa cells promotes cell proliferation and affects the cell cycle.

### RASAL2 promotes the PCa cell cycle through upregulation of cyclin D1

**Fig. 3:**
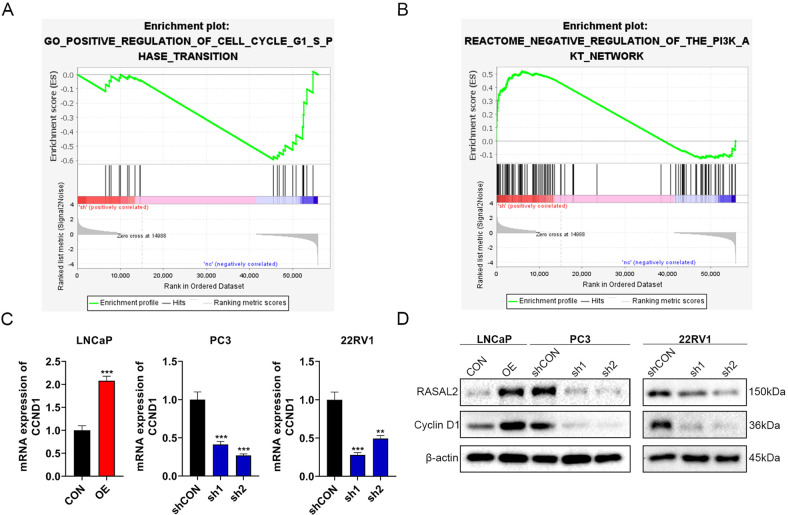
Effect of RASAL2 on the PI3K/AKT pathway and cyclin D1 (CCND1) expression. **A**, **B** GSEA for RNA sequencing of PCa sublines with RASAL2 knockdown. **C**, **D** Quantitative real-time RT–PCR and Western blotting analysis of CCND1 mRNA expression in established cell lines.

To explore the signalling pathways by which RASAL2 affects cell proliferation, we performed RNA sequencing on a RASAL2-knockdown 22RV1 cell line. GSEA revealed that RASAL2 knockdown suppressed the G1-S phase transition and negatively regulated the PI3K/AKT network (Fig. 3A, B), in which cyclin D1, D3 and E1 were identified to be involved in RASAL2-induced cell cycle transitions. Cell cycle progression is strictly manipulated by cyclins and their related regulatory factors, and cyclin D1 is one of the major cell cycle proteins that play a key role in the G1-S phase transition. The cyclin D1 gene (CCND1) has been demonstrated to be upregulated in PCa and is associated with cancer progression and poor prognosis in PCa patients [16–18]. To determine whether a regulatory relationship exists between RASAL2 and cyclin D1, expression levels of CCND1 in several PCa sublines were detected by Western blot and RT–qPCR (Fig. 3C, D). We noted that cyclin D1 was upregulated in the RASAL2-overexpressing LNCaP subline but downregulated in the PC3 and 22RV1-RASAL2-knockdown sublines compared to controls, consistent with expectations.

### RASAL2 promotes PCa cell proliferation via the PI3K/AKT/cyclin D1 signalling pathway

**Fig. 4:**
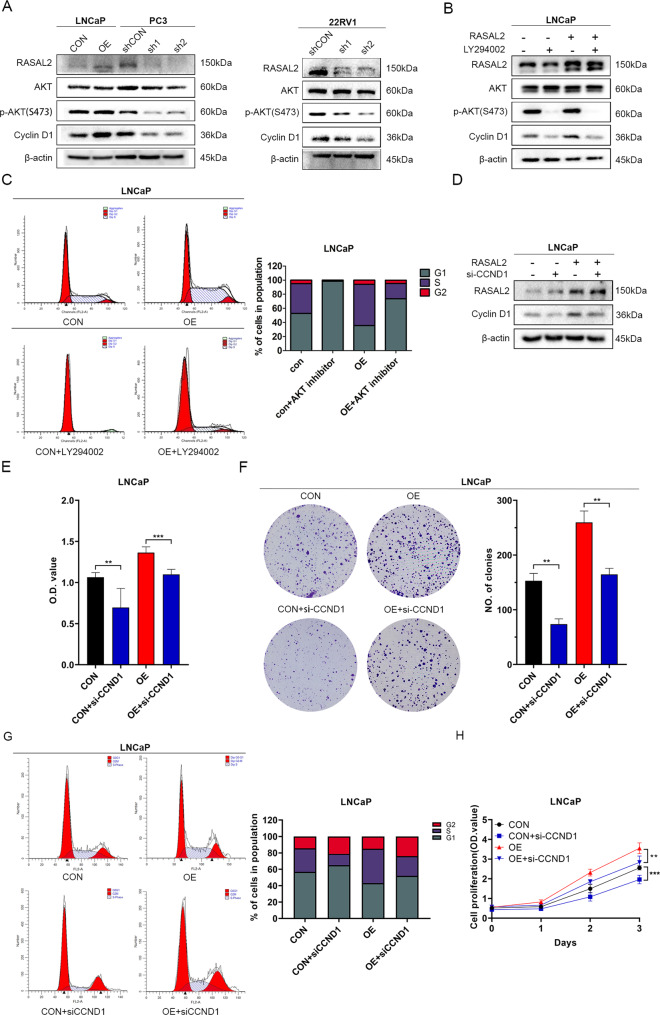
RASAL2 regulates PCa cell proliferation via PI3K/AKT/cyclin D1 signalling. **A** Western blotting analysis of AKT, p-AKT and cyclin D1 expression levels in established cell lines. **B** Western blotting analysis of AKT, p-AKT and cyclin D1 expression in the LNCaP cell line treated with an AKT inhibitor. **C** Flow cytometry analysis of AKT inhibitor-treated LNCaP cell lines. **D** Western blotting analysis of cyclin D1 expression levels in the siCCND1-treated LNCaP cell line. Cell viability (**E**), colony formation (**F**), cell cycle (**G**) and cell proliferation (**H**) assays in siCCND1-treated LNCaP cell lines.

Furthermore, we attempted to elucidate the mechanism of cyclin D1 regulation by RASAL2. In GSEA, we found that the knockdown of RASAL2 negatively regulated the PI3K/AKT network (Fig. 3B). The PI3K/AKT pathway is one of the classical pathways associated with cell proliferation and has been shown to be associated with cell proliferation in a variety of tumours [19]. Additionally, PI3K/AKT signalling was found to be one of the common genomically altered pathways in mCRPC patients [2]; in particular, a previous study suggested that RASAL2 promotes cancer cell proliferation through this pathway in hepatocellular carcinoma [20]. To determine the mechanisms by which RASAL2 regulates the PI3K/AKT signalling pathway and cyclin D1 expression, we examined protein expression levels of total and phosphorylated AKT in PCa sublines with RASAL2 overexpression or knockdown. Indeed, RASAL2 altered phosphorylation levels of AKT (Fig. 4A and Supplemental Fig. 2). Therefore, we treated LNCaP/OE sublines with a specific inhibitor of the PI3K/AKT pathway (LY294002). Cyclin D1 was significantly downregulated in response to treatment with the inhibitor (Fig. 4B), while changes in the cell cycle caused by RASAL2 overexpression were rescued by the decrease of p-AKT (Fig. 4C). These results suggested that RASAL2 could affect cyclin D1 expression by regulating the level of phosphorylated AKT.

Next, to clarify the positive regulatory effects of RASAL2 on PCa cell proliferation through upregulation of cyclin D1 expression, we reduced cyclin D1 expression in LNCaP sublines using siRNA (Fig. 4D). We then assayed the cell viability and proliferation levels of siCCND1-treated LNCaP cell lines. Indeed, siCCND1 reduced the enhancement of cellular activity caused by the upregulation of RASAL2, which in turn significantly affected cell proliferation and cell cycle distribution (Fig. 4E–H). All of these data suggest that RASAL2 regulates the PCa cell cycle through PI3K/AKT/cyclin D1 and subsequently affects PCa cell proliferation.

### RASAL2 promotes tumorigenicity and cyclin D1 expression in vivo

**Fig. 5:**
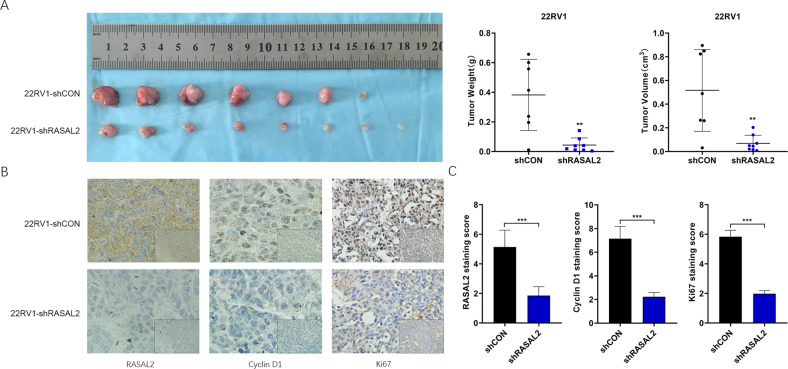
RASAL2 modulates PCa tumour growth and cyclin D1 expression in vivo. **A** Images of the growth of subcutaneous xenografts established by control and RASAL2 knockdown cells and statistical results of tumour mass and volume. **B**, **C** Imaging and quantitative results of immunohistochemical analysis of expression levels of RASAL2, cyclin D1 and Ki67 in transplanted tumours.

To further determine whether RASAL2 affects PCa cell proliferation in vivo, we used a subcutaneous xenograft model established with 22RV1/KD sublines. Knockdown of RASAL2 decreased tumour volume and weight compared to the control group (Fig. 5A). Similar results were also observed in the PC3 cell model (data not shown). Immunohistochemical staining of the xenograft tissues showed that the protein expression levels of RASAL2 and cyclin D1 were significantly decreased in the knockdown group compared to controls, consistent with the in vitro findings. In addition, the expression of Ki67 in the knockdown group was lower than that in the control group (Fig. 5B, C). These data demonstrate that RASAL2 promotes PCa tumour growth and cyclin D1 expression in vivo.

### RASAL2 and cyclin D1 are positively correlated in PCa specimens

**Fig. 6:**
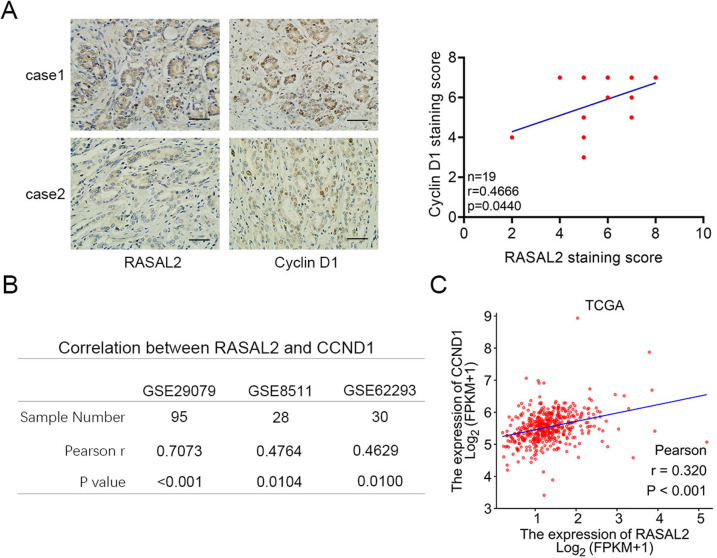
Correlation of RASAL2 and cyclin D1 in clinical specimens. **A** Immunohistochemical analysis of the expression levels and correlation of RASAL2 and cyclin D1 in clinical samples of PCa (*n* = 19). **B** Molecular correlation analysis of RASAL2 and cyclin D1 (CCND1) from the GEO database (GSE29079, GSE8511 and GSE62293). **C** Correlation analysis of RASAL2 and cyclin D1 (CCND1) from TCGA database.

To strengthen our findings regarding the relationship between RASAL2 and cyclin D1, we assessed their expression levels in clinical samples of PCa and observed a positive correlation between them (Fig. 6A). At the same time, analysis of TCGA and GEO datasets (GSE29079, GSE8511 and GSE69223) further supported our finding (Fig. 6B, C). Together, these results indicate a positive correlation between RASAL2 and cyclin D1 in PCa and indicate that RASAL2 acts as an oncogene in PCa progression.

## Discussion

An unlimited and uncontrolled ability to proliferate is the defining characteristic of cancer, including prostate adenocarcinoma. Currently, the primary treatment for PCa is ADT, which is based on the unique dependence of PCa on the AR signalling axis [21]. ADT, a cornerstone of AR antagonists or docetaxel chemotherapy, has significantly improved the 5-year survival rate of PCa patients [3]. However, this treatment strategy becomes limited when the disease progresses to CRPC, and cross-resistance also compromises the long-term efficacy of drugs targeting AR signalling, including second-generation anti-androgens [2]. Therefore, the need to identify additional drugs or therapeutic targets for PCa growth is urgent. RASAL2 is a member of the RAS-GAP family, but previous studies have revealed that its role in different tumours is inconsistent [6]. In the present study, we demonstrated that RASAL2 was an oncogene in PCa that promoted PCa cell proliferation and tumour growth through the PI3K/AKT/cyclin D1 signalling pathway.

RAS proteins are small-molecule GTPases that play a key role in biological activities, including cell proliferation, survival, differentiation, motility and gene transcription. In several human tumour types, RAS acts through its GTPase activity, while mutations in RAS family genes will cause significant effects in a subset of tumours [22]. GTPase-activating proteins (GAPs) are key regulators of RAS proteins that play diverse roles in cell signalling [5]. RASAL2 is a unique member of this family; initially, RASAL2 was identified as a potential tumour suppressor in a cell-based screen developed by Min et al. [23]. McLaughlin et al. first reported that RASAL2 played an antitumour role in breast cancer and that its deletion exerted a causal effect on breast cancer progression and metastasis [24]. Subsequent studies have reported its suppressive effects in luminal B breast cancer, ovarian cancer, pancreatic ductal carcinoma, nasopharyngeal carcinoma and malignant astrocytoma [6, 7]. However, with extensive research, RASAL2 was found to be upregulated in triple-negative and oestrogen-receptor negative breast cancers and was associated with tumour invasion, metastasis, and poor prognosis [8–10]. Among them, Feng et al. found that manipulation of RASAL2 in two highly invasive TNBC cell lines (i.e. MB-231-LN and BT549) could not induce a change in RAS GTP level. Furthermore, they found that GAP domain deletion mutants but not GAP activity-deficient mutants of RASAL2 could exert carcinogenic effects, indicating that the oncogenic effect of RASAL2 may not depend on the catalytic activity of GAP. And the role of RASAL2 in colorectal, lung and liver cancers has also emerged as controversial [6, 11, 12]. Notably, this difference in the RASAL2 function is thought to be related to its phosphorylation. Wang et al. found that phosphorylation levels of RASAL2 at S237 were a determinant of its function, and they found that phosphorylation levels of RASAL2 were higher in ER-negative breast tumour cells than in ER-positive cells in vivo and in vitro, subsequently affecting its function [25]. Bao et al. then reported that RASAL2 had the same biological effect on autophagy in triple-negative and ER-positive breast cancer cells and that glucose starvation induced RASAL2 phosphorylation at S351, transforming its function from an inhibitor to an activator of AMPK-mediated autophagy, which in turn affected tumour progression [26]. In our previous study, we reported that RASAL2 acted as a tumour suppressor in renal cancer and bladder cancer [13–15]. However, we found that RASAL2 was an oncogene that was significantly overexpressed in PCa tissues compared to normal tissues and was correlated with clinical stage, Gleason score and poor patient prognosis. Through functional studies conducted in vitro and in vivo, we demonstrated that RASAL2 promoted cell proliferation and tumour growth in PCa. In our established PCa cell lines, we observed that RASAL2 might be involved in regulating the PI3K/AKT pathway and affected the G1-S phase transition in tumour cells. Also, we tried to explore the phosphorylation of RASAL2 in PCa and predicted rich potential phosphorylation sites of RASAL2 (data not shown). In terms of gene mutations, previous publications and databases have revealed that the mutation of KRAS and RASAL2 in PCa were infrequent events and did not correlate significantly with clinicopathological features.

Cyclin D1 (CCND1) represents one of the most frequently amplified loci among all human cancer types and plays a key role in the transition of cells from the G1 to S phase [4, 27]. Cyclin D1 has been reported to promote cancer progression in a variety of cancers [28–30]. In PCa, cyclin D1 is involved in the regulation of cell growth, migration, invasion, apoptosis and neuroendocrine differentiation [16–18]. In the present study, cyclin D1 is one of the most important cyclins identified from the GSEA results in the G1-S phase transition regulated by RASAL2, which is highly correlated in the TCGA-PRAD database (Supplemental Fig. 3). Notably, cyclin D1 is a downstream effector of the PI3K/AKT pathway [31, 32], one of the critical pathways that promote tumour development [19, 33, 34]. In PCa, the PI3K/AKT pathway is one of the common genomically altered pathways in mCRPC patients due to PTEN loss and maybe a potential target for alternative therapy for PCa that is not sensitive to anti-androgen therapy. Interestingly, targeting both the PI3K and AR pathways significantly inhibited tumour growth in preclinical models of PTEN resistance and enzalutamide resistance, suggesting potential crosstalk between the pathways and demonstrating the potential for combination therapeutic strategies [2]. Inhibitors targeting the cell cycle and PI3K/AKT pathway are being tried for the treatment of mCRPC; in fact, trials using the AKT inhibitor ipatasertib in combination with abiraterone and prednisone are already underway, and capivasertib with enzalutamide has produced therapeutic responses in patients with PTEN deficiency or AKT activating mutations. In addition, other inhibitors of the AKT pathway have also been evaluated. With respect to the cell cycle, palbociclib, a selective inhibitor of CDK4/CDK6, has been used as a monotherapy and in combination with AR pathway inhibitors for the treatment of mCRPC [3]. For RASAL2, Mclaughlin reported elevated p-AKT levels in a mouse model of RASAL2-silenced mammary carcinoma [24]; however, Fang found that inhibition of RASAL2 in hepatocellular carcinoma was followed by a decrease in p-AKT levels [20], and our previous study demonstrated that RASAL2 regulated bladder cancer angiogenesis through p-AKT. All of these results suggest a direct relationship between RASAL2 and p-AKT. Based on this, our study revealed that RASAL2 regulated the levels of cyclin D1 through phosphorylation of AKT, promoting cell proliferation of PCa cells by affecting cell cycle distribution. To further rule out the mechanism of RASAL2 regulating cyclin D1, we performed RNA sequencing-based on our RASAL2 overexpression and knockdown PCa cell models and co-analyzed with the predicted results of cyclin D1 upstream transcription factors obtained from Animal TFDB (http://bioinfo.life.hust.edu.cn/AnimalTFDB/), and found several potential transcription factors which might be responsible for RASAL2-induced cyclin D1 expression (Supplemental Fig. 4).

In summary, we have revealed the expression and role of RASAL2 in prostate cancer for the first time. We provided evidence that RASAL2 may specifically promote the proliferation of PCa cells through the PI3K/AKT/cyclin D1 signalling pathway, which identified RASAL2 as a potential prognostic marker and therapeutic target for PCa.

## Materials and methods

### Cell culture and reagents

All PCa cell lines (LNCaP, C4-2, 22RV1, PC3 and DU145) were obtained from the American Type Culture Collection (ATCC, Manassas, VA, USA). Cells were cultured in RPMI-1640 medium (Sigma, USA) with 10% foetal bovine serum (Biological Industries, USA), penicillin (100 U/ml) and streptomycin (0.1 mg/ml) at 37 °C and 5% CO_2_.

### Cell viability assay

LNCaP, PC3 and 22RV1 cells were seeded into 96-well plates at 5000 cells per well. After culturing for 48 h, the media was removed, and 180 μl fresh media mixed with 20 μl MTT (Sigma–Aldrich; USA) was added to each well for 4 h. Then, the media was removed completely, and 150 μl DMSO per well was added, followed by shaking for 10 min. Finally, the OD value was determined using a microplate spectrophotometer (BioTek, Epoch, USA).

### Clone formation assay

Clone formation assays were used to assess the in vitro clonogenicity capabilities of established cell lines. Cells in the exponential growth phase were collected, and an equal number of cells were seeded into 6-well plates for at least two weeks to form colonies, with fresh medium changes every 3–4 days. Then, each well was washed two times with PBS, fixed in 4% paraformaldehyde, stained with 0.1% crystalline violet solution for 20 min at room temperature, and finally washed with distilled water to remove the excess dye. The number of colonies was quantified for each sample.

### Cell proliferation experiment

After digestion and centrifugation, fresh media containing 10% foetal bovine serum was added to cells in the logarithmic growth phase, which were mixed and then seeded into 96-well plates at 200 µl/well (approximately 5000 cells/well). Sterile PBS was added to the surrounding wells to prevent excessive evaporation of the medium, after which the plates were gently transferred to the cell incubator and cultured overnight. Four 96-well plates, in the same way, At least 3 h after cell inoculation, the medium was carefully aspirated, 100 µl of fresh medium and 10 µl of CCK8 was added to each well, mixed well and incubated for 4 h at 37 °C. Then, the OD value at 450 nm was measured using a microplate spectrophotometer (BioTek, Epoch, USA). The other 96-well plates were determined using the same method when the incubation was continued for 24, 48 and 72 h.

### Flow cytometry analysis of the cell cycle

Treated LNCaP or PC3 cell sublines were cultured in 6-cm culture dishes, and the cells were collected during the exponential growth phase, washed with PBS, and stained with the cell cycle kit according to the manufacturer’s instructions, and immediately analyzed by flow cytometry.

### Real-time PCR

All RNA was extracted using an RNA Fast 200 kit (Feijie Biotechnology, Shanghai, China) and quantified in a spectrophotometer. Reverse transcription of RNA was performed using a Prime Script RT–PCR kit (Takara Bio Dalian, China). Then, a CFX96 Real-Time PCR system (Bio-Rad, CA, USA) was used to assess the RASAL2 and CCND1 gene expression levels. The RASAL2 primer sequence was “F: ACAGACACAGCAGGTTCAGT; R: AGCAAGCGGCGTTCATATTC”. The CCND1 primer sequence was “F: TCCCACTCCTACGATACGCT; R: CAGGGCCGTTGGGTAGAAAA”. The primer sequence for 18 S was “F: GGAATTGACGGAAGGGCACCACC; R: GTGCAGCCCCGGACATCTAAGG” and was used as a control. Finally, the RASAL2 and CCND1 mRNA expression levels were quantified using the 2^−ΔΔCT^ method and are shown as fold changes compared to the control.

### Western blotting analysis

All cells were seeded into six-well plates 24 h before protein extraction. Then, RIPA buffer containing proteinase inhibitors was used to lyse the cells. Western blot membranes were blocked in Tris-buffered saline with 0.1% Tween 20 and 5% skim milk for 1 h. After blocking, primary antibodies against RASAL2 (CST, #82481, 1:1000), PI3K (CST, #4249, 1:1000), AKT (CST, #5741, 1:1000), p-AKT (S473, D9E, 1:1000) or cyclin D1 (WL01435a, 1:1000) were incubated with the membranes overnight at 4 °C. After the membranes were washed three times with TBST, they were incubated with horseradish peroxidase-conjugated secondary antibodies for 1 h and visualised using an ECL chemiluminescent detection system (Bio-Rad Laboratories, Inc.). A monoclonal anti-β-actin antibody (CST, #3700) was used to normalise loading differences. Experiments were repeated three times.

### RASAL2 overexpression and knockdown

The RASAL2 short hairpin RNA (shRNA) plasmid LVRU6GP was purchased from Genecopoeia (Guangzhou, China) and was used to package the lentiviruses with the PAX2 and PMDG2 plasmids in 293 T cells according to a previously published protocol. For overexpression of RASAL2, the lentiviral system EX-E2664-Lv201 (Genecopoeia, Guangzhou, China) was used. LNCaP cell lines were transfected with overexpression lentiviruses, and the PC3 and 22RV1 cell lines were used to establish RASAL2-knockdown cell lines.

### Transient knockdown of CCND1

CCND1 siRNA was purchased from GenePharma. Referring to the manufacturer’s instructions, LNCaP control and RASAL2-overexpressing cell sublines were cultured in 6-cm dishes and transfected with siCCND1 during the exponential growth phase.

### Subcutaneous xenograft tumour model

A RASAL2-knockdown 22RV1 cell line was used to establish a subcutaneous tumour model. Eight 4-week-old male nude mice were randomly divided into two groups without blinding, and 8 × 10^6^ cells were injected into both sides in 150 µl of serum-free RPMI-1640 medium. All nude mice were sacrificed, and tumours were collected after 4 weeks.

### Clinical specimens and immunohistochemical analysis

To examine the expression of RASAL2 in PCa tissues and its correlation with tumour growth, cancer tissues with adjacent nontumor tissues and matched metastatic lymph node tissues from 19 patients with prostate cancer were obtained from the Department of Urology, The First Affiliated Hospital of Xi’an Jiaotong University. All specimens were used only after written consent was obtained from the patients. IHC was performed using immunohistochemistry kits (Gene Tech, Shanghai). Briefly, tissue sections were deparaffinized, rehydrated and subjected to 0.01 M citrate buffer, microwave treated at medium-high temperature for 30 min and then naturally cooled to room temperature. Then, endogenous peroxidase and alkaline phosphatase activities were incubated in a blocking solution for 40 min. After blocking in 5% BSA for 30 min, sections were incubated with antibodies against RASAL2 (Genetex, C2C3, 1:200), cyclin D1 (WL01435a, 1:200) or Ki67 (WL01384a, 1:200) overnight at 4 °C. Then, secondary antibodies were added and incubated at room temperature for 30–40 min, followed by staining with a diaminobenzidine (DAB) kit and haematoxylin, dehydration and sealing of the sections for evaluation.

Results were evaluated according to the intensity of the staining (0, 1+, 2+ and 3+) and the percentage of positive cells, which were classified as 0 (0%), 1 (1–25%), 2 (26–50%), 3 (51–75%) and 4 (76–100%). Finally, the staining scores and staining levels were analysed together to derive the combined results: negative (0 points), weak (1–4 points), moderate (5–8 points) and strong (9–12 points).

### RNA sequencing and gene set enrichment analysis (GSEA)

22RV1 cell lines with RASAL2 knockdown were used for RNA sequencing by GENEWIZ (www.genewiz.com). Next-generation sequencing library preparations were constructed according to the manufacturer’s protocol (NEBNext^®^ Ultra™ RNA Library Prep Kit for Illumina^®^). GSEA software was used to analyse the pathways.

### Bioinformatics and statistical analysis

GEO, TCGA and Oncomine data were used for bioinformatics analysis. Public datasets (GSE68555, GSE29079, GSE8511 and GSE62293) were downloaded from the NCBI GEO database (November 2020). All statistical analyses were performed using GraphPad Prism version 8.0 software (GraphPad Software, CA, USA). All error bars in the graphical data represent the mean ± SD, and differences between the two groups were tested using a two-tailed Student’s *t*-test. *p* < 0.05 was considered statistically significant.

## Supplementary information

supplemental information

supplemental Figure 1

supplemental Figure 2

supplemental Figure 3

supplemental Figure 4

original western blots-Fig2A RASAL2

original western blots-Fig2A beta-actin

original western blots-Fig2C 22RV1-RASAL2

original western blots-Fig2C 22RV1-beta-actin

original western blots-Fig2C LNCaP-RASAL2

original western blots-Fig2C LNCaP-beta-actin

original western blots-Fig3D 22RV1-RASAL2

original western blots-Fig3D 22RV1-cyclin D1

original western blots-Fig3D 22RV1-beta-actin

original western blots-Fig3D LNCaP-RASAL2

original western blots-Fig3D LNCaP-cyclin D1

original western blots-Fig3D LNCaP-beta-actin

original western blots-Fig4A 22RV1-AKT

original western blots-Fig4A 22RV1-RASAL2

original western blots-Fig4A 22RV1-cyclin D1

original western blots-Fig4A 22RV1-pAKT

original western blots-Fig4A 22RV1-beta-actin

original western blots-Fig4A LNCaP-AKT

original western blots-Fig4A LNCaP-RASAL2

original western blots-Fig4A LNCaP-cyclin D1

original western blots-Fig4A LNCaP-pAKT

original western blots-Fig4A LNCaP-beta-actin

original western blots-Fig4B LNCaP-AKT

original western blots-Fig4B LNCaP-RASAL2

original western blots-Fig4B LNCaP-cyclin D1

original western blots-Fig4B LNCaP-pAKT

original western blots-Fig4B LNCaP-beta-actin

original western blots-Fig4D-RASAL2

original western blots-Fig4D-cyclin D1

original western blots-Fig4D-beta-actin

## Data Availability

All the data used during the study are available from the corresponding author on request.
