# Supplementary material for: RASAL2 regulates the cell cycle and cyclin D1 expression through PI3K/AKT signalling in prostate tumorigenesis
Source: Cell Death Discov. 2022 Jun 6;8:275. doi: 10.1038/s41420-022-01069-3 (PMC9170709; doi:10.1038/s41420-022-01069-3)
Supplement: Supplementary file 1 — supplemental information [file 41420_2022_1069_MOESM1_ESM.docx]

**Supplemental Figure 1. Expression of RASAL2 in PCa tissues and correlation with overall survival of patients.**

A, Immunohistochemical analysis of RASAL2 expression in prostate cancer and normal adjacent tissues under the same field of view. B, Overall survival of PCa patients associated with RASAL2 expression derived from the TCGA database

**Supplemental Figure 2. Western blotting analysis of pAkt (T308) in established cell lines.**

**Supplemental Figure 3. Correlation analysis of three potential downstream cyclins (cyclin D1, D3 and E1) with RASAL2 in TCGA-PRAD database.**

**Supplemental Figure 4. Predicted results of transcription factors that may be involved in the RASAL2-inducted cyclin D1 expression.**
