# Supplementary figures and images for: RASAL2 regulates the cell cycle and cyclin D1 expression through PI3K/AKT signalling in prostate tumorigenesis
Source: Cell Death Discov. 2022 Jun 6;8:275. doi: 10.1038/s41420-022-01069-3 (PMC9170709; doi:10.1038/s41420-022-01069-3)

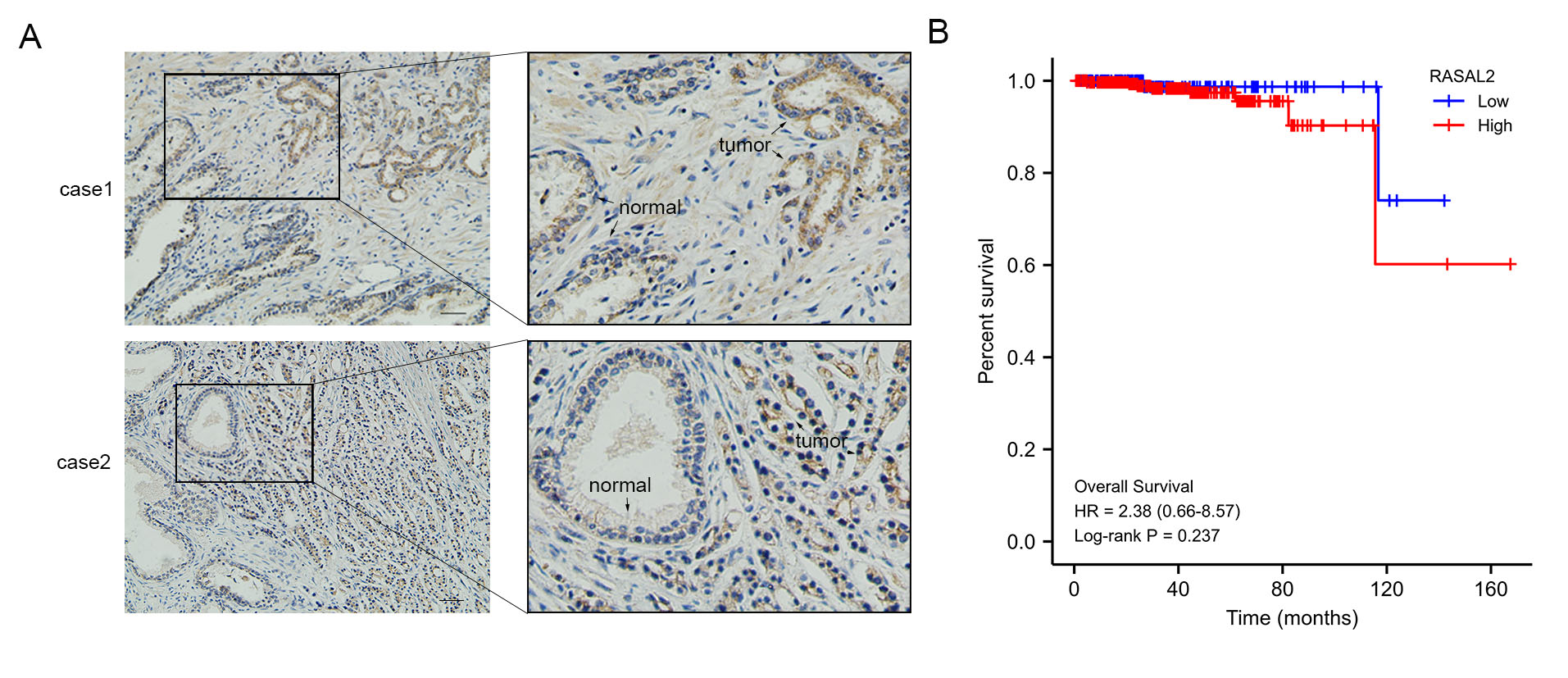

Supplement: Supplementary file 2 — supplemental Figure 1 [file 41420_2022_1069_MOESM2_ESM.jpg]

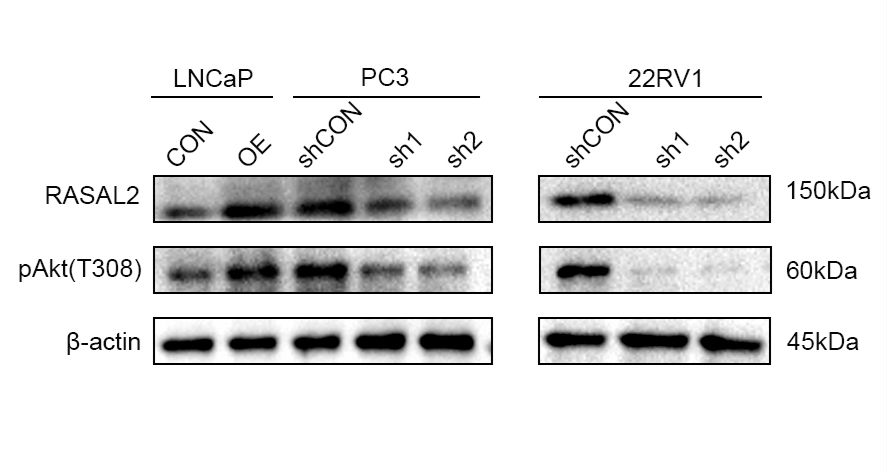

Supplement: Supplementary file 3 — supplemental Figure 2 [file 41420_2022_1069_MOESM3_ESM.jpg]

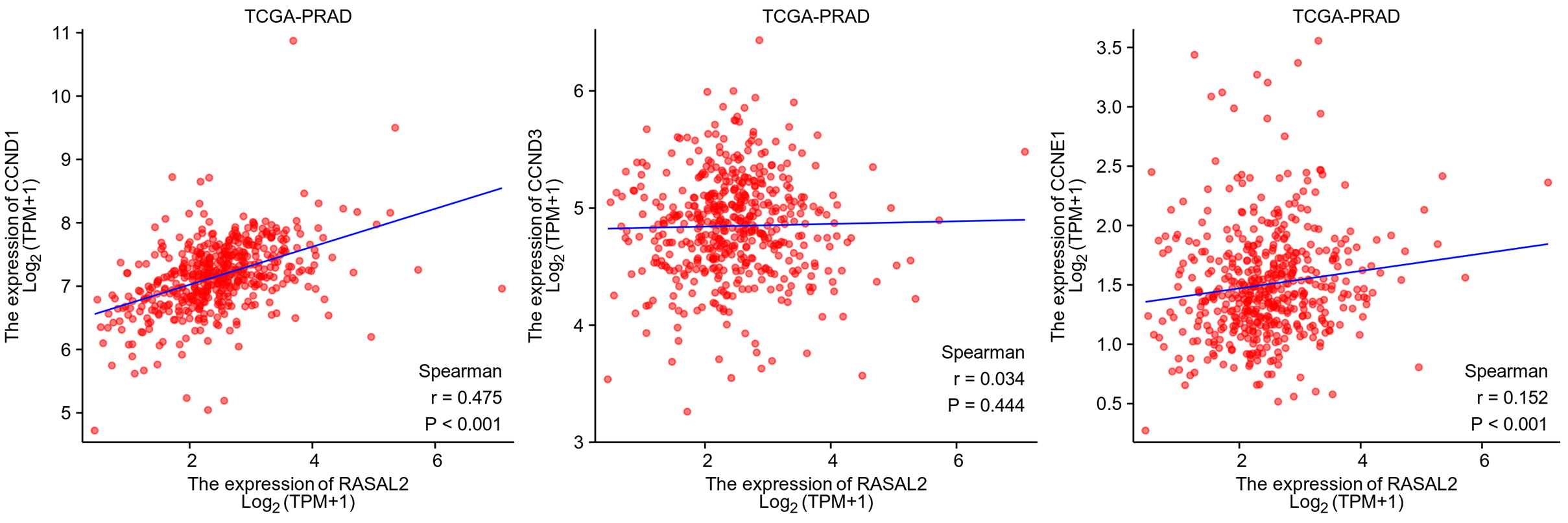

Supplement: Supplementary file 4 — supplemental Figure 3 [file 41420_2022_1069_MOESM4_ESM.jpg]

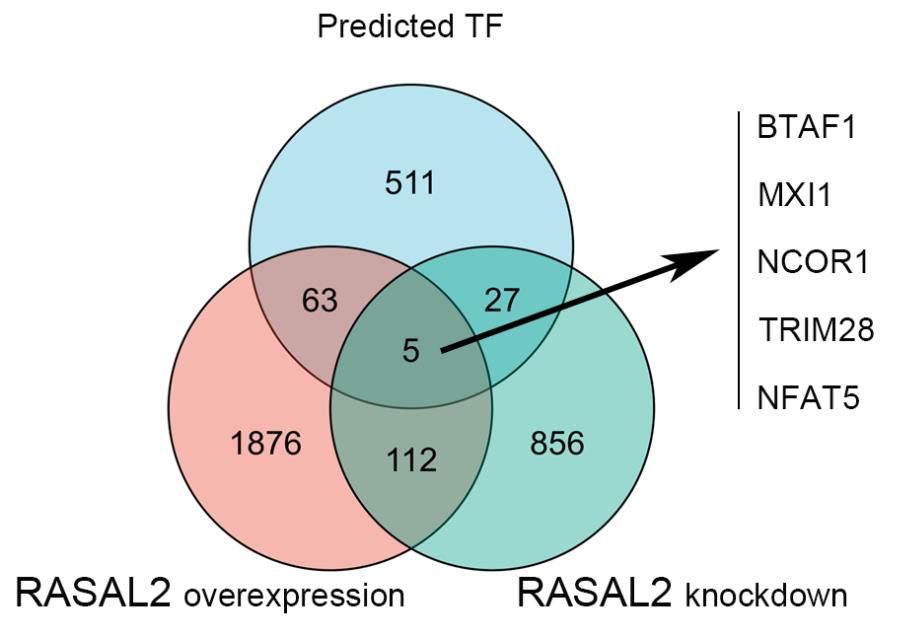

Supplement: Supplementary file 5 — supplemental Figure 4 [file 41420_2022_1069_MOESM5_ESM.jpg]

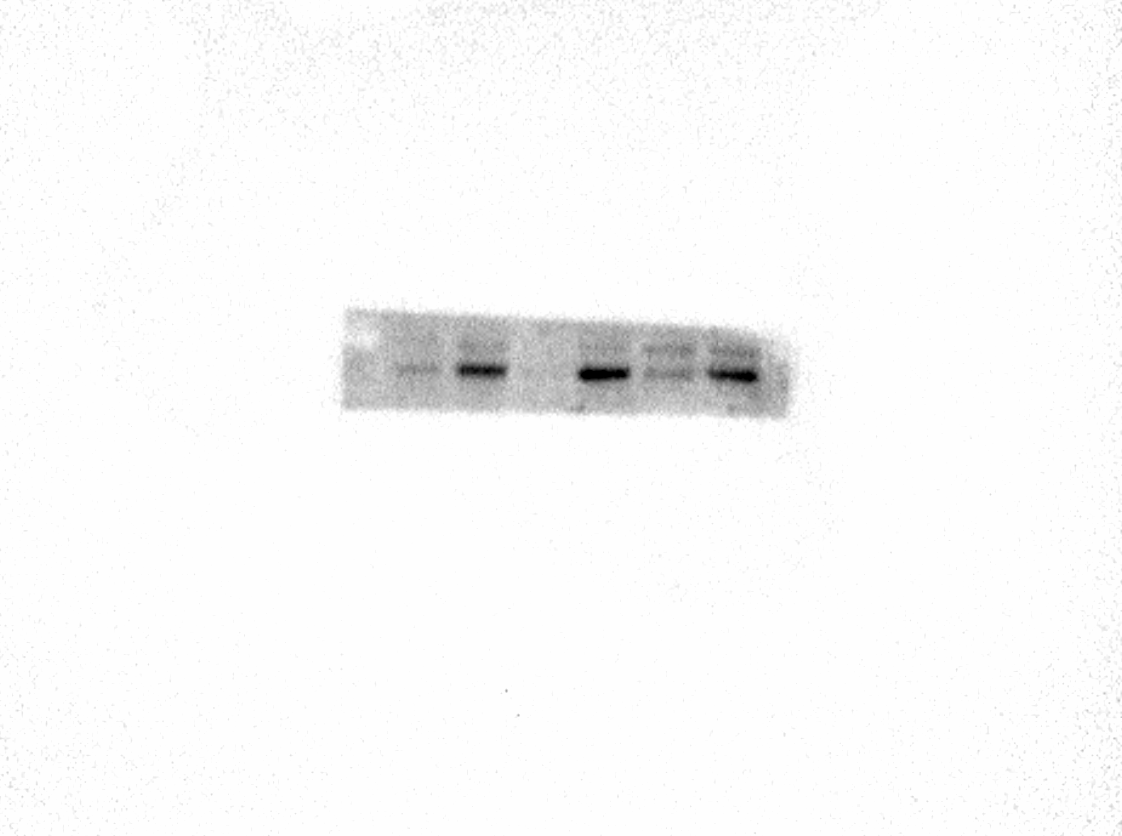

Supplement: Supplementary file 6 — original western blots-Fig2A RASAL2 [file 41420_2022_1069_MOESM6_ESM.tif]

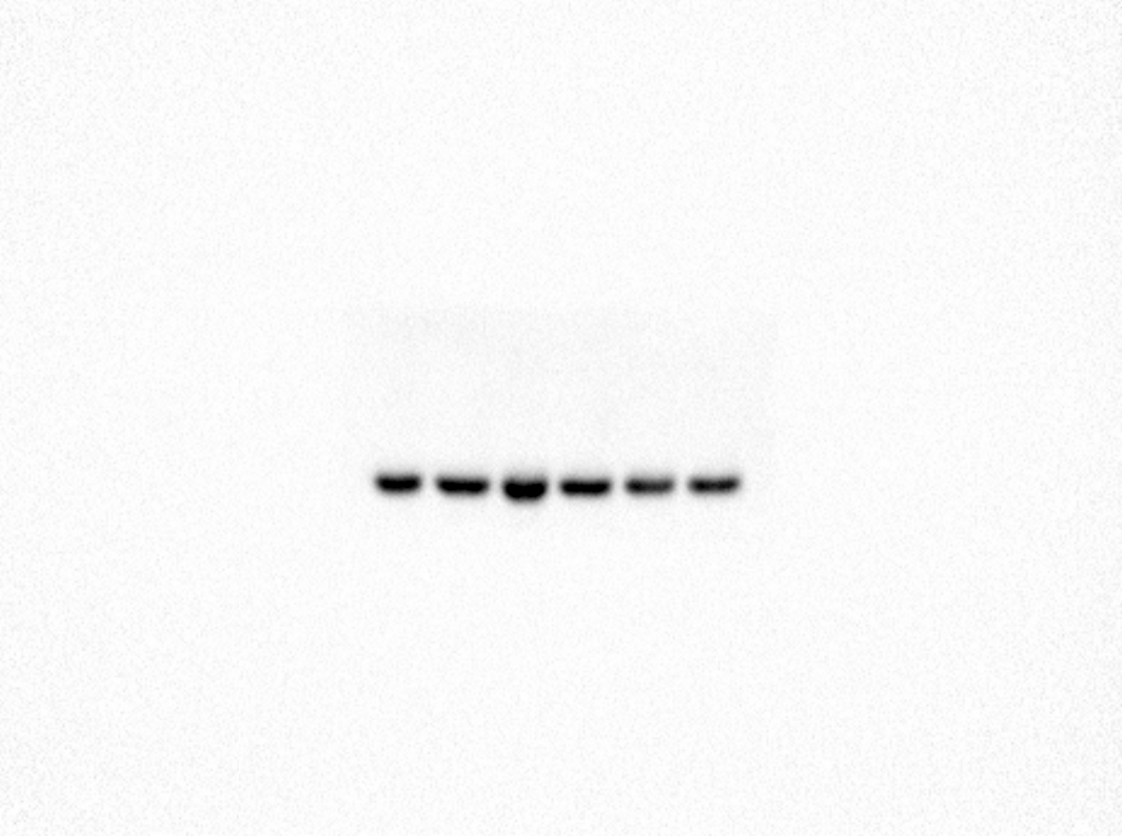

Supplement: Supplementary file 7 — original western blots-Fig2A beta-actin [file 41420_2022_1069_MOESM7_ESM.tif]

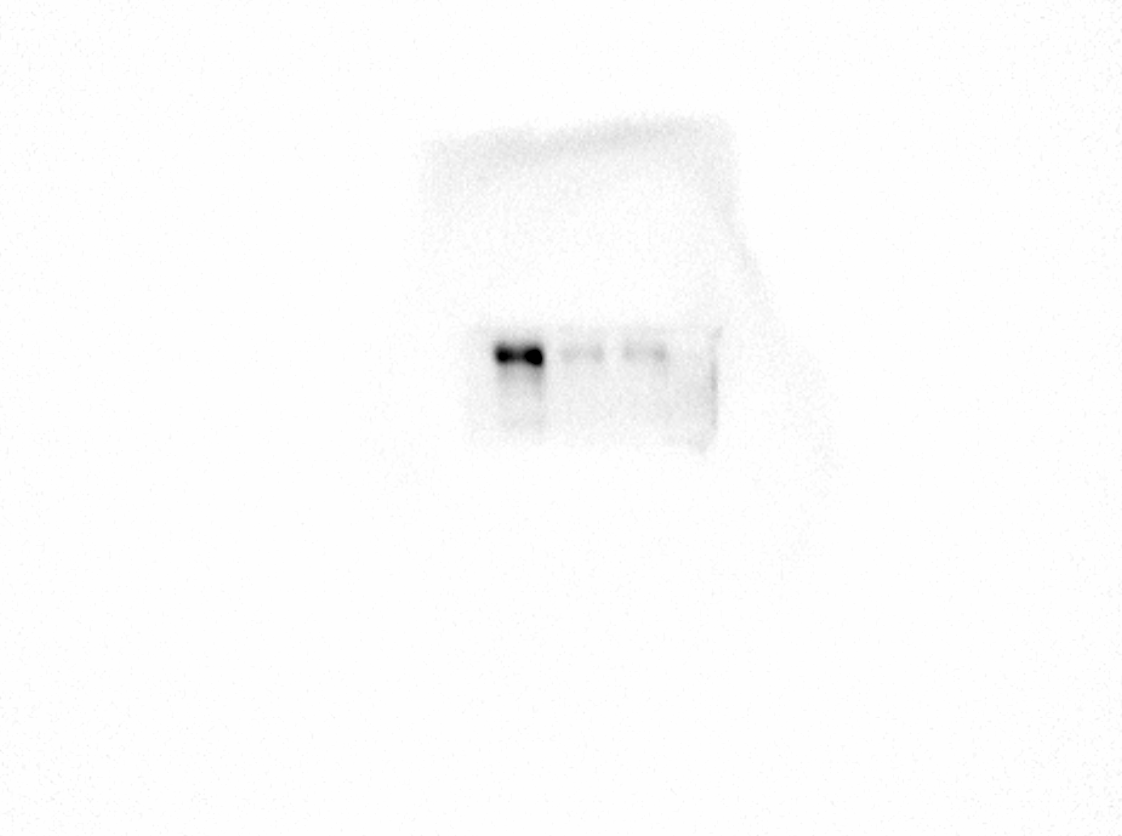

Supplement: Supplementary file 8 — original western blots-Fig2C 22RV1-RASAL2 [file 41420_2022_1069_MOESM8_ESM.tif]

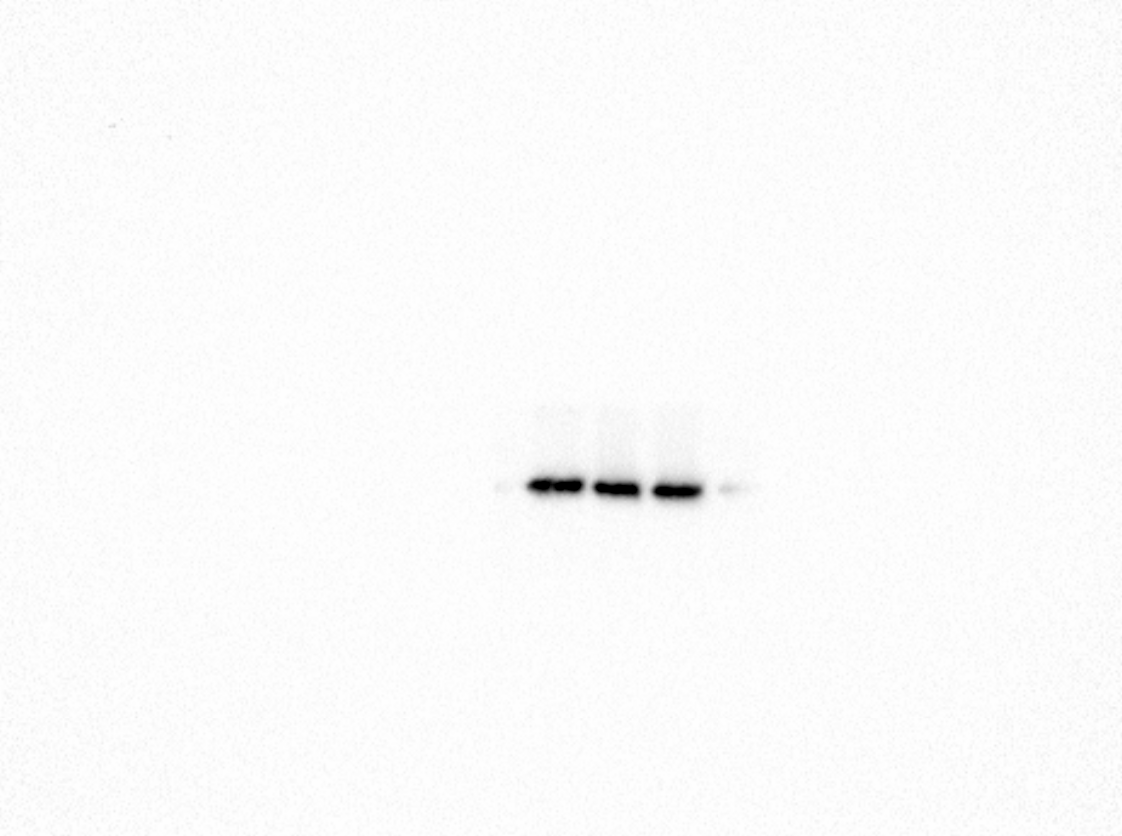

Supplement: Supplementary file 9 — original western blots-Fig2C 22RV1-beta-actin [file 41420_2022_1069_MOESM9_ESM.tif]

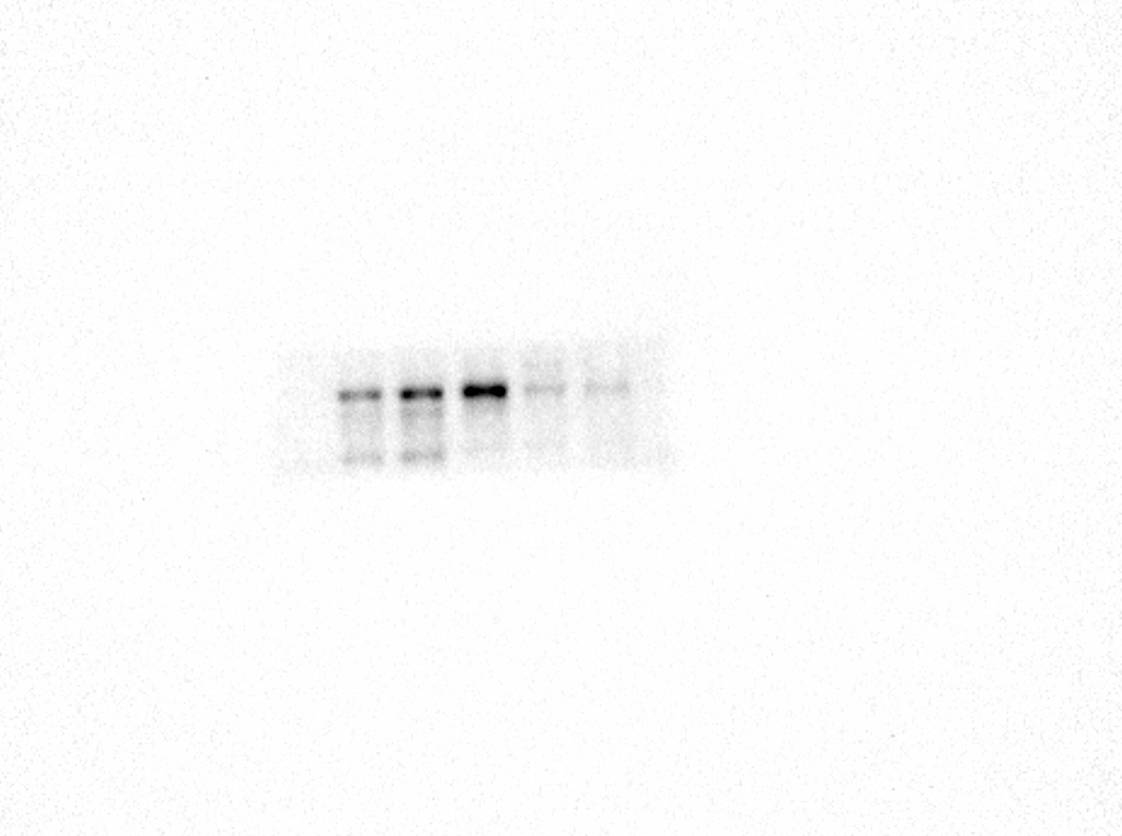

Supplement: Supplementary file 10 — original western blots-Fig2C LNCaP-RASAL2 [file 41420_2022_1069_MOESM10_ESM.tif]

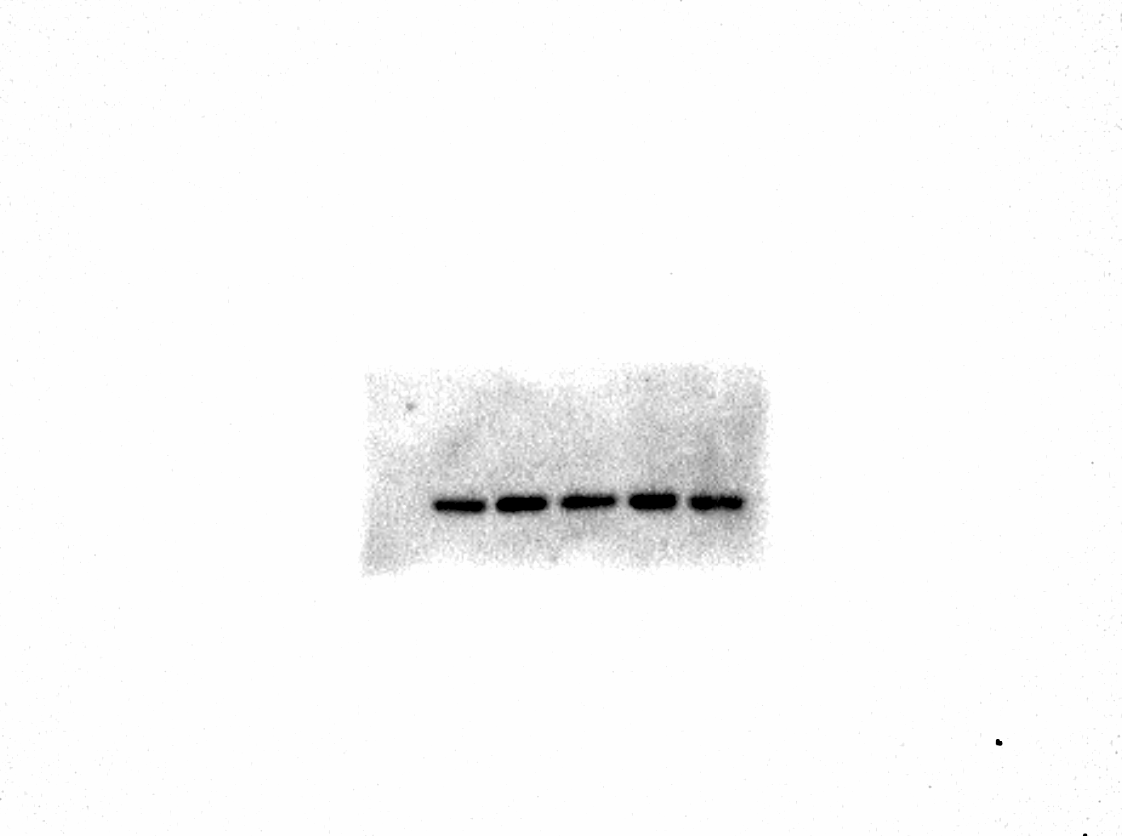

Supplement: Supplementary file 11 — original western blots-Fig2C LNCaP-beta-actin [file 41420_2022_1069_MOESM11_ESM.tif]

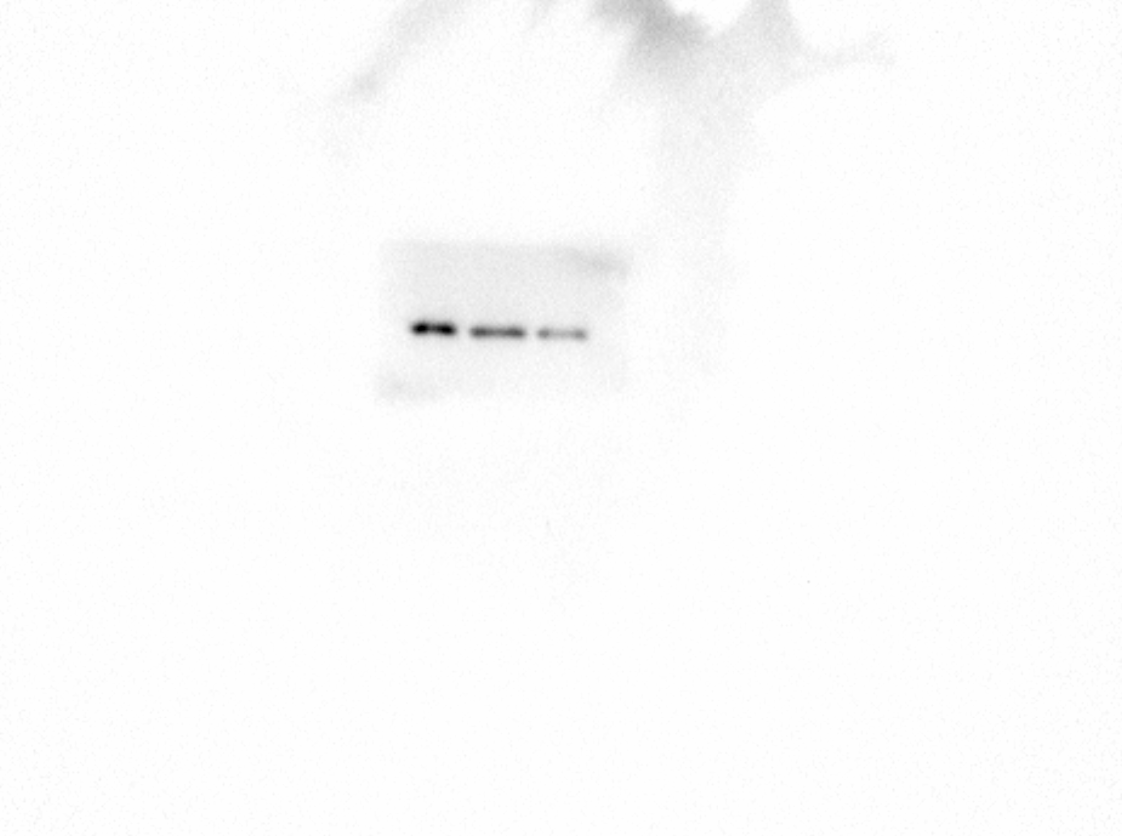

Supplement: Supplementary file 12 — original western blots-Fig3D 22RV1-RASAL2 [file 41420_2022_1069_MOESM12_ESM.tif]

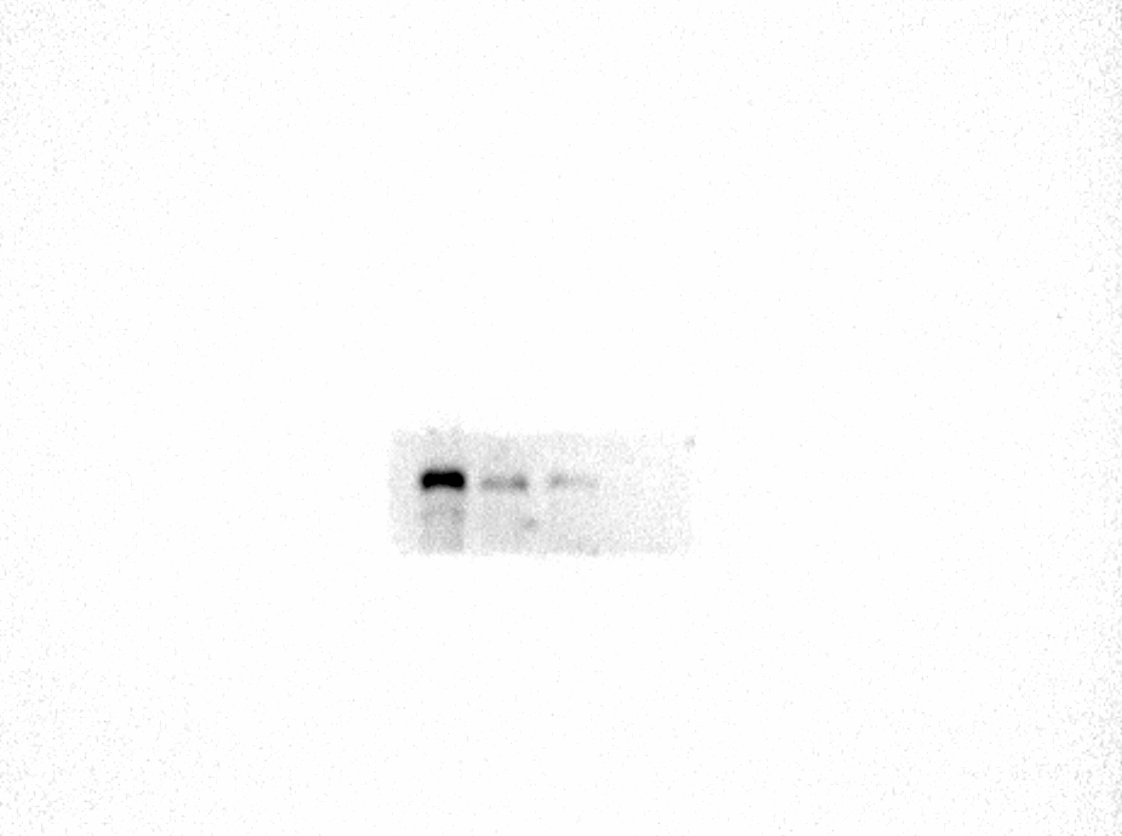

Supplement: Supplementary file 13 — original western blots-Fig3D 22RV1-cyclin D1 [file 41420_2022_1069_MOESM13_ESM.tif]

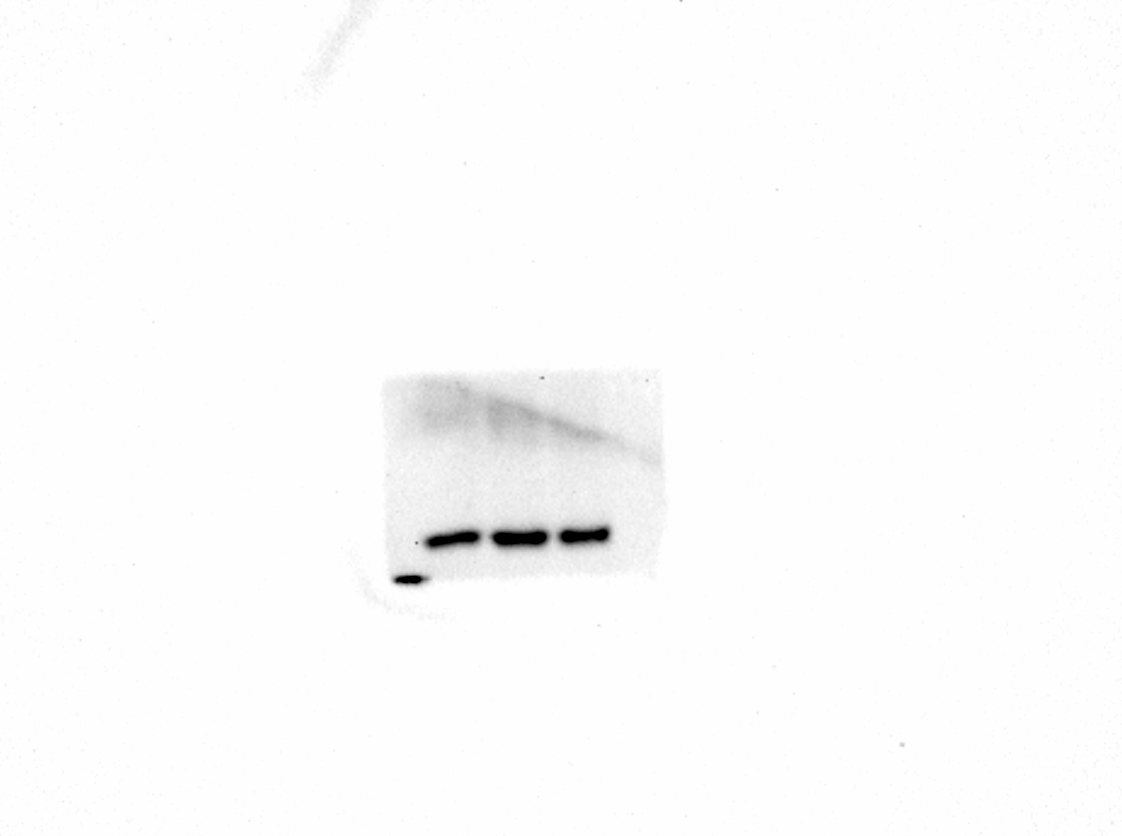

Supplement: Supplementary file 14 — original western blots-Fig3D 22RV1-beta-actin [file 41420_2022_1069_MOESM14_ESM.tif]

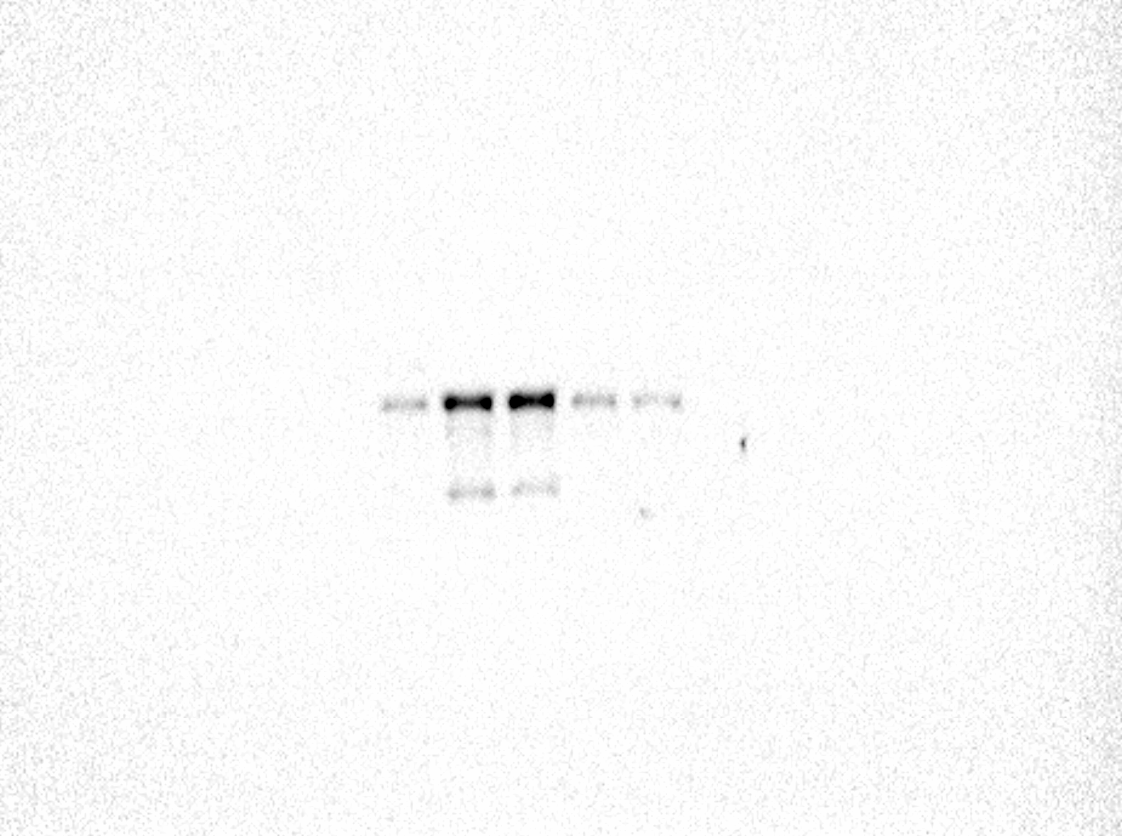

Supplement: Supplementary file 15 — original western blots-Fig3D LNCaP-RASAL2 [file 41420_2022_1069_MOESM15_ESM.tif]

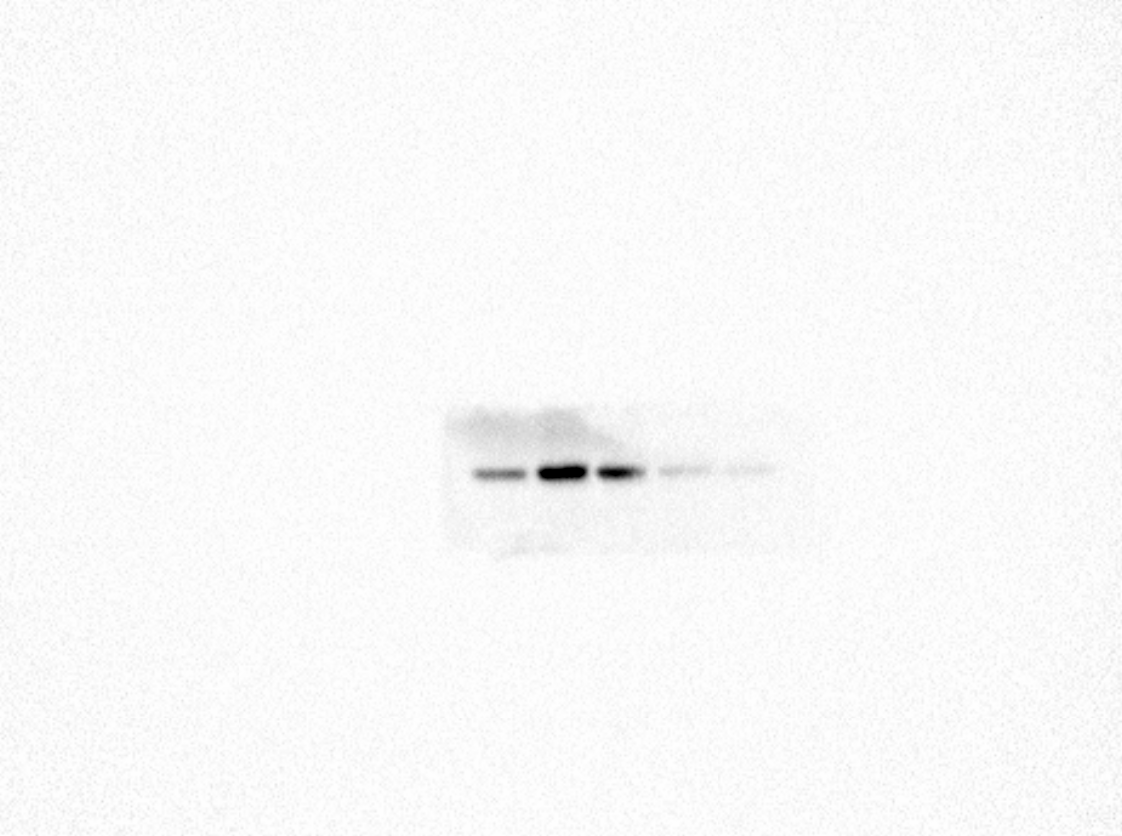

Supplement: Supplementary file 16 — original western blots-Fig3D LNCaP-cyclin D1 [file 41420_2022_1069_MOESM16_ESM.tif]

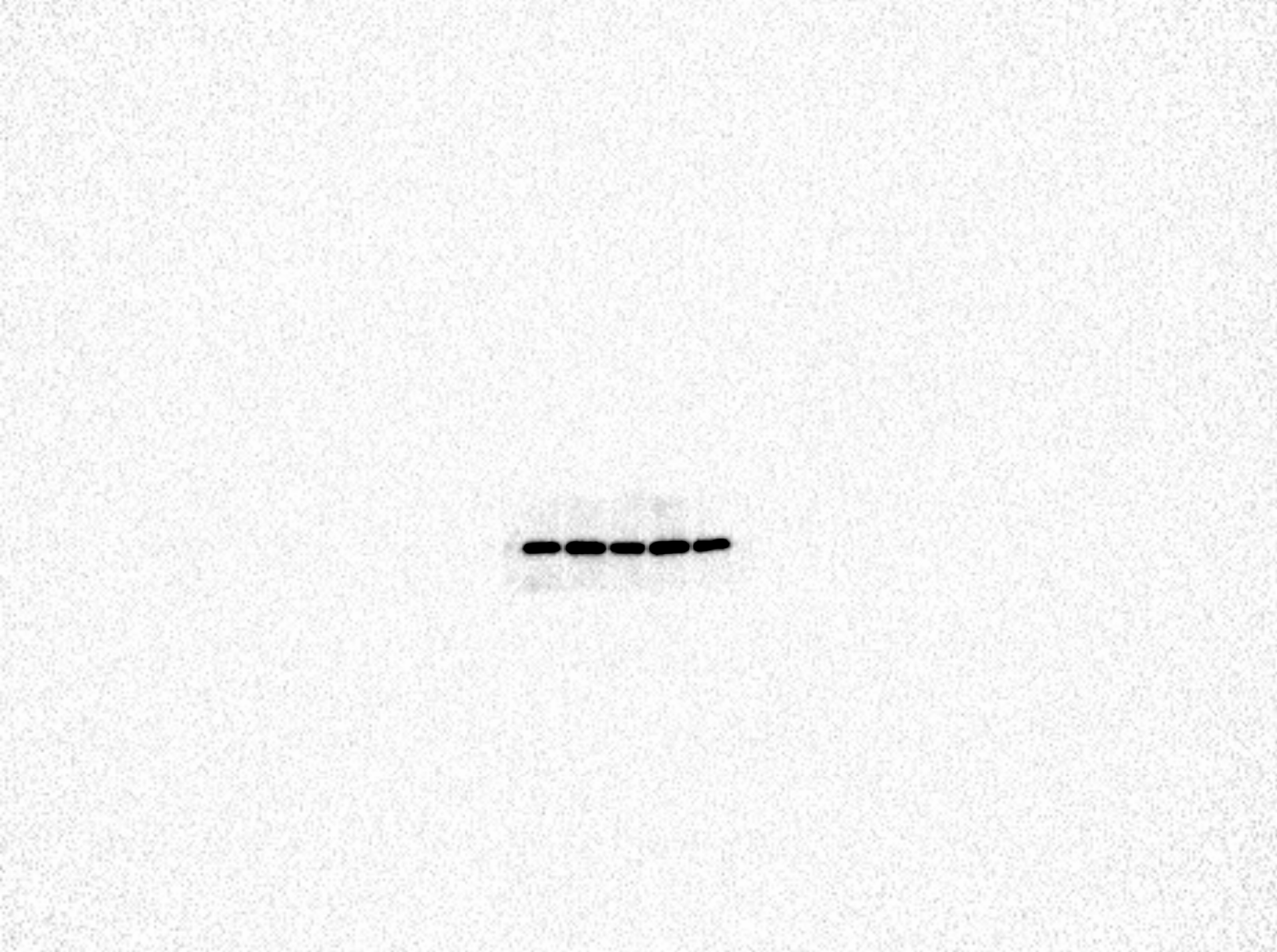

Supplement: Supplementary file 17 — original western blots-Fig3D LNCaP-beta-actin [file 41420_2022_1069_MOESM17_ESM.tif]

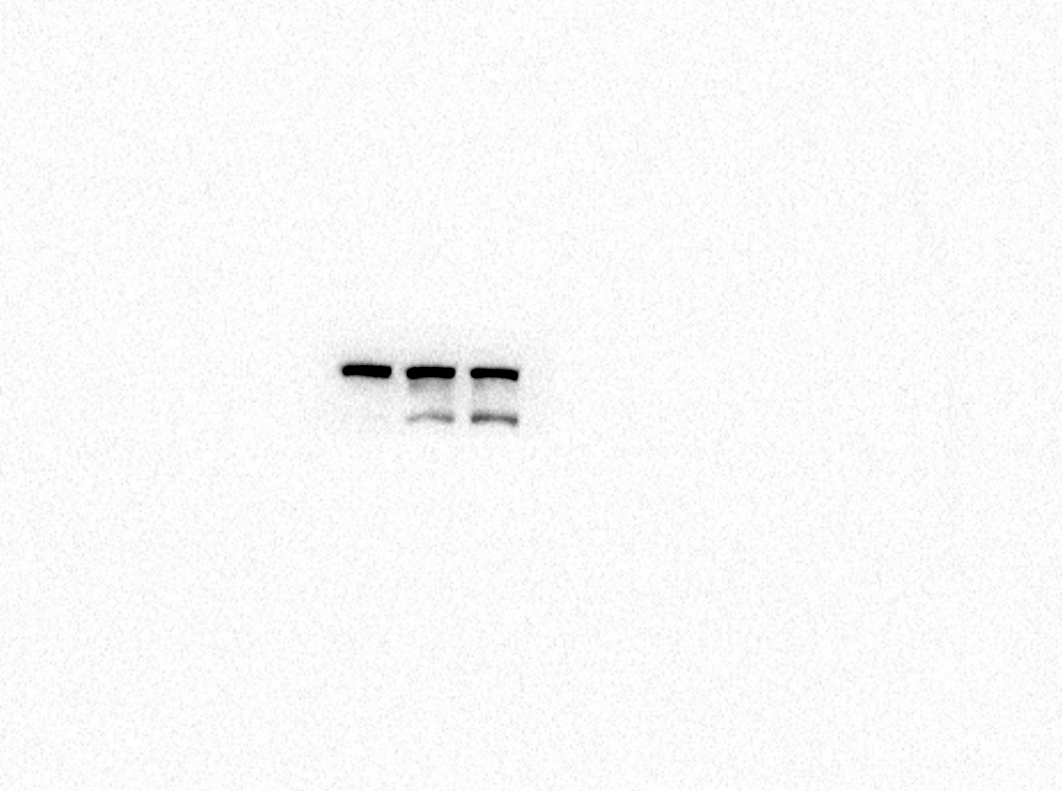

Supplement: Supplementary file 18 — original western blots-Fig4A 22RV1-AKT [file 41420_2022_1069_MOESM18_ESM.tif]

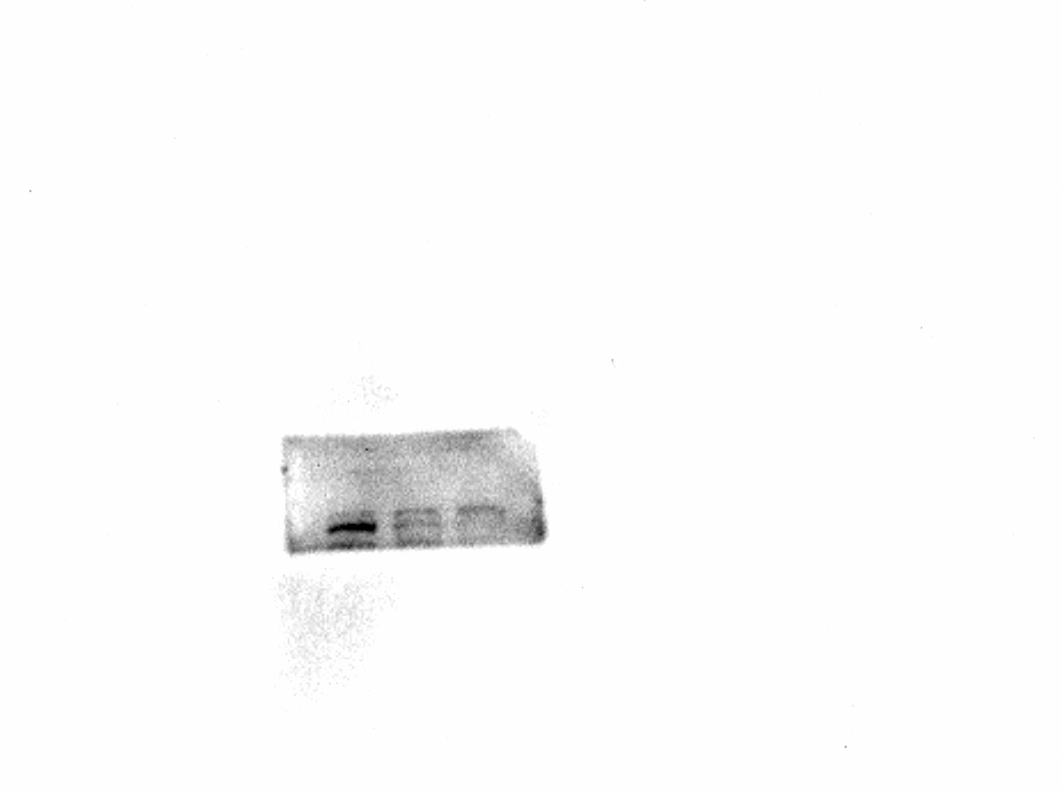

Supplement: Supplementary file 19 — original western blots-Fig4A 22RV1-RASAL2 [file 41420_2022_1069_MOESM19_ESM.tif]

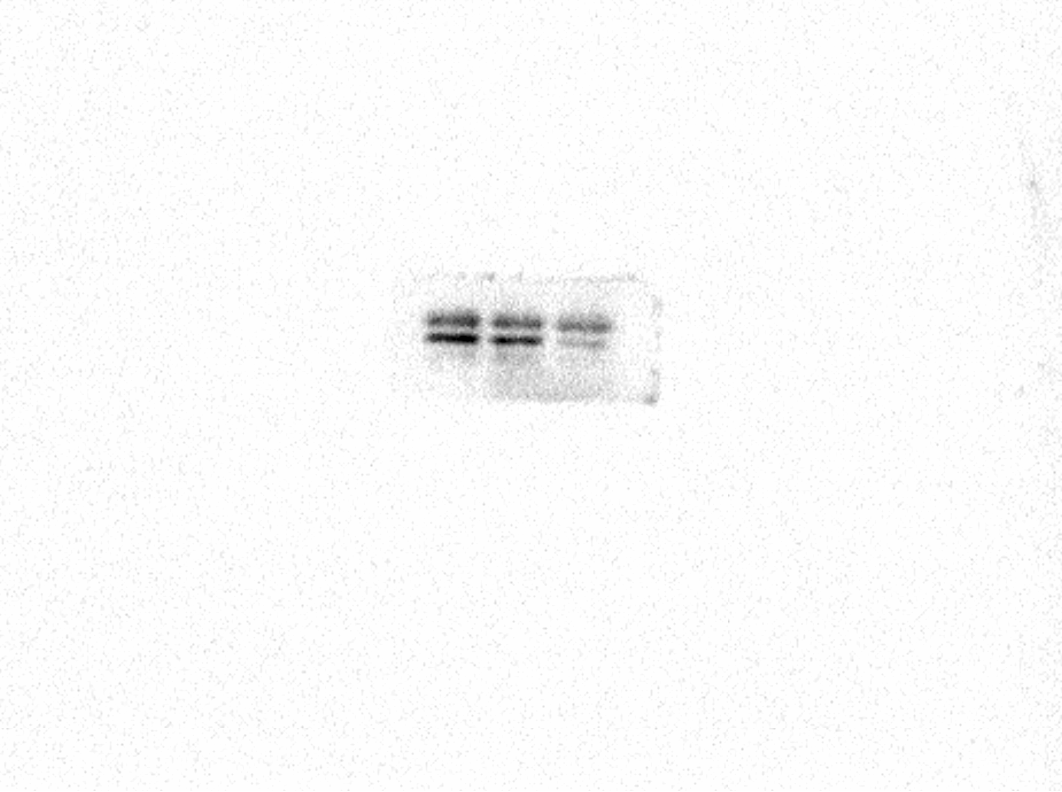

Supplement: Supplementary file 20 — original western blots-Fig4A 22RV1-cyclin D1 [file 41420_2022_1069_MOESM20_ESM.tif]

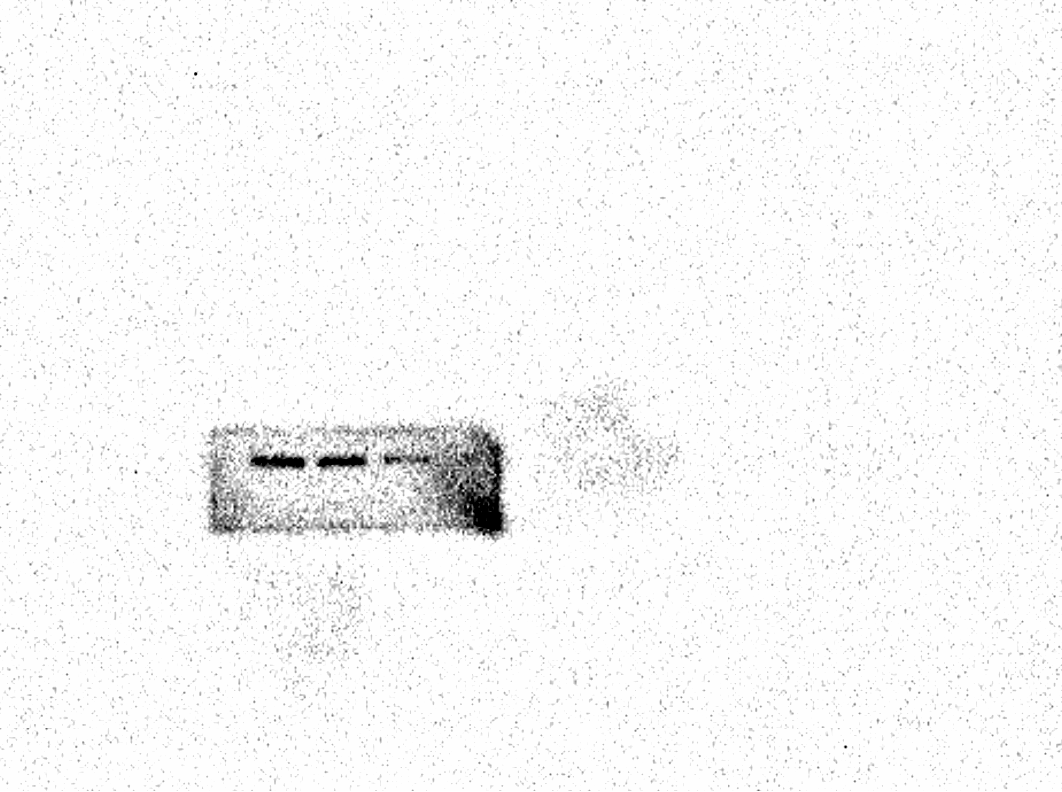

Supplement: Supplementary file 21 — original western blots-Fig4A 22RV1-pAKT [file 41420_2022_1069_MOESM21_ESM.tif]

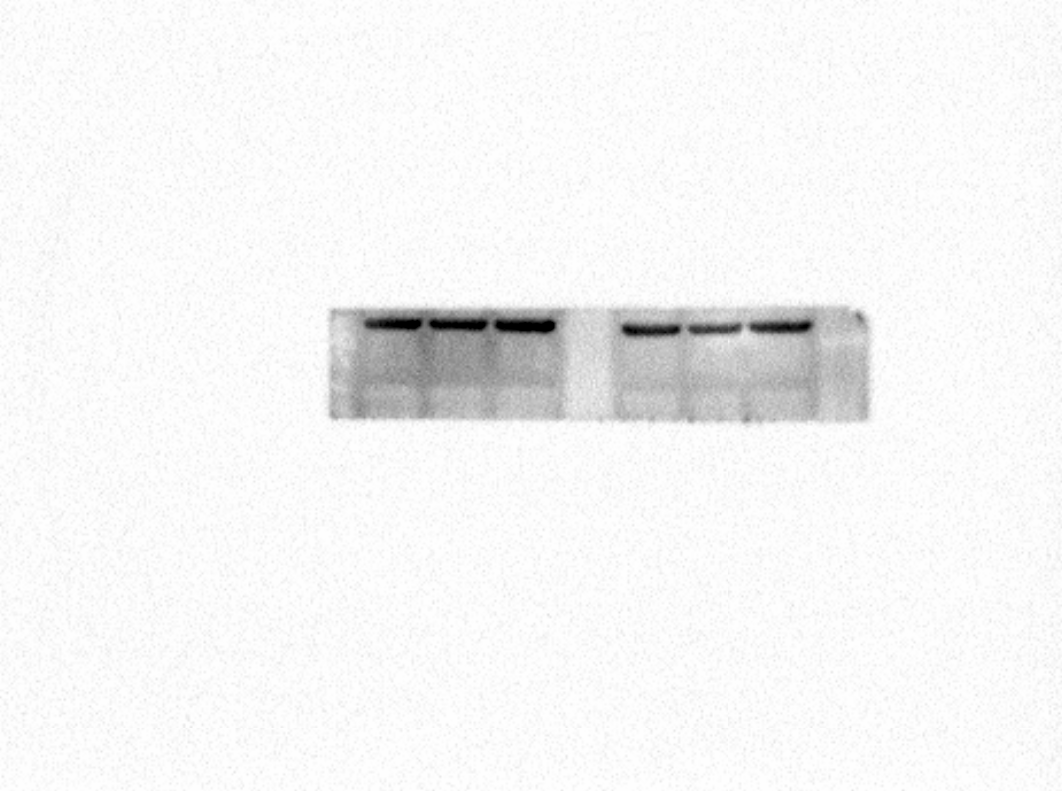

Supplement: Supplementary file 22 — original western blots-Fig4A 22RV1-beta-actin [file 41420_2022_1069_MOESM22_ESM.tif]

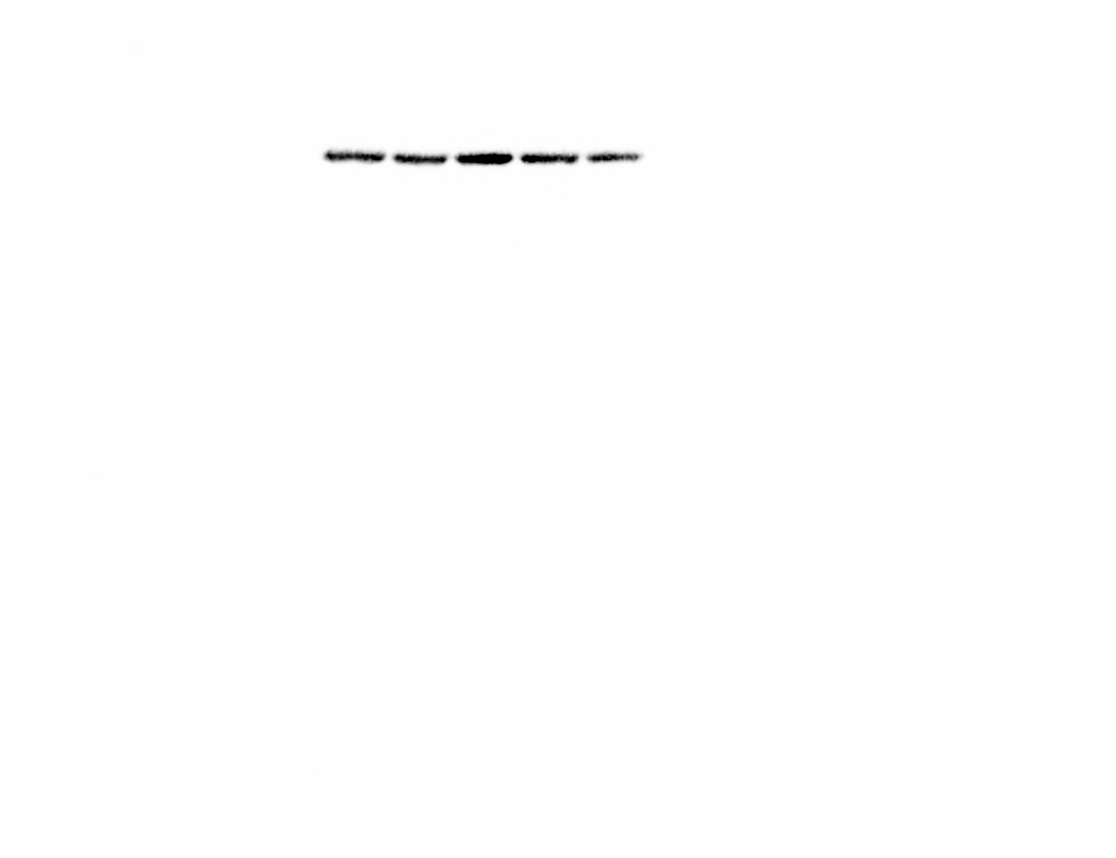

Supplement: Supplementary file 23 — original western blots-Fig4A LNCaP-AKT [file 41420_2022_1069_MOESM23_ESM.tif]

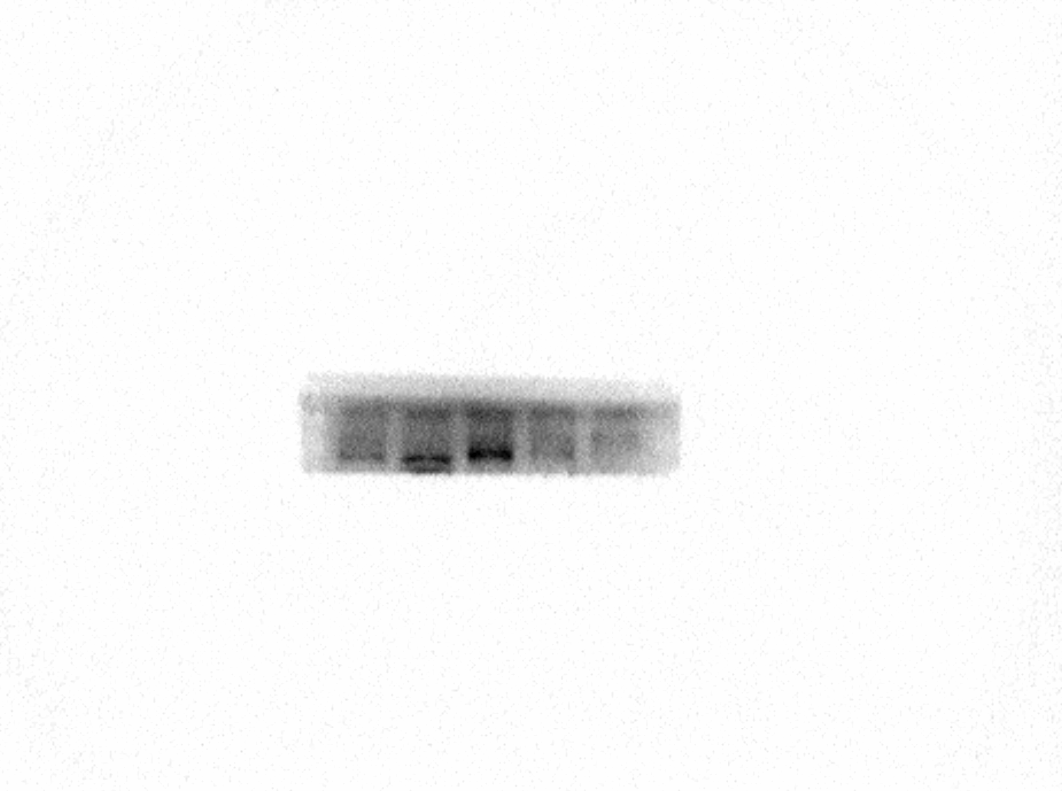

Supplement: Supplementary file 24 — original western blots-Fig4A LNCaP-RASAL2 [file 41420_2022_1069_MOESM24_ESM.tif]

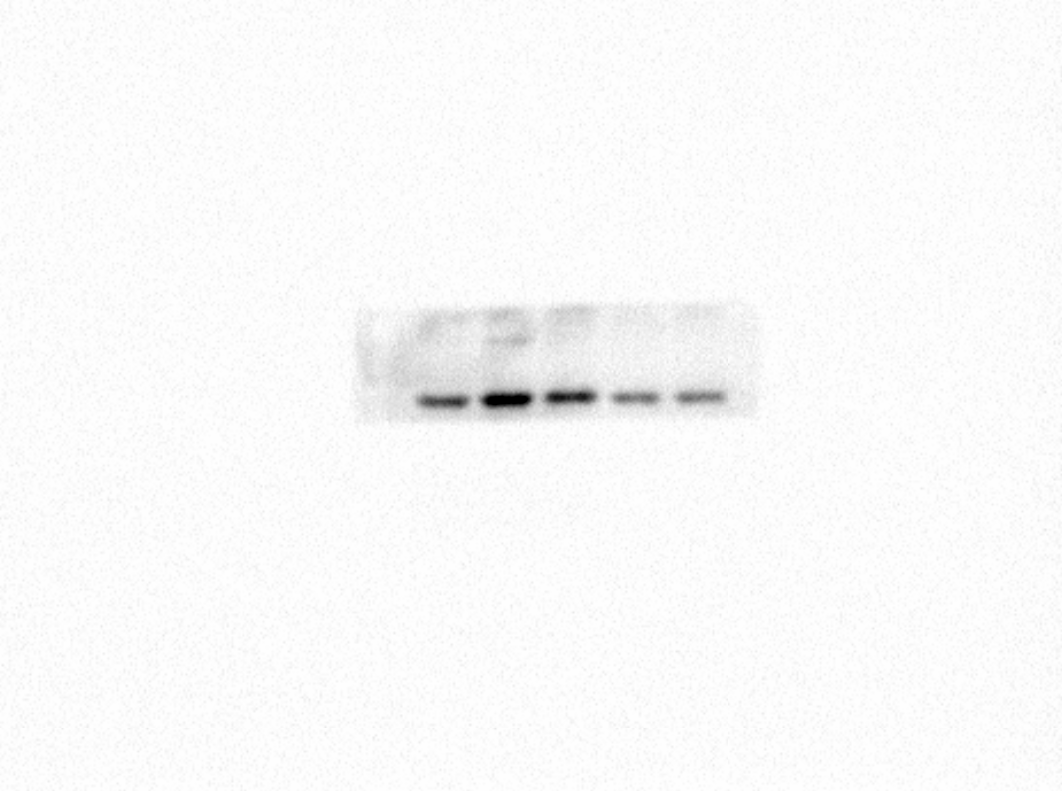

Supplement: Supplementary file 25 — original western blots-Fig4A LNCaP-cyclin D1 [file 41420_2022_1069_MOESM25_ESM.tif]

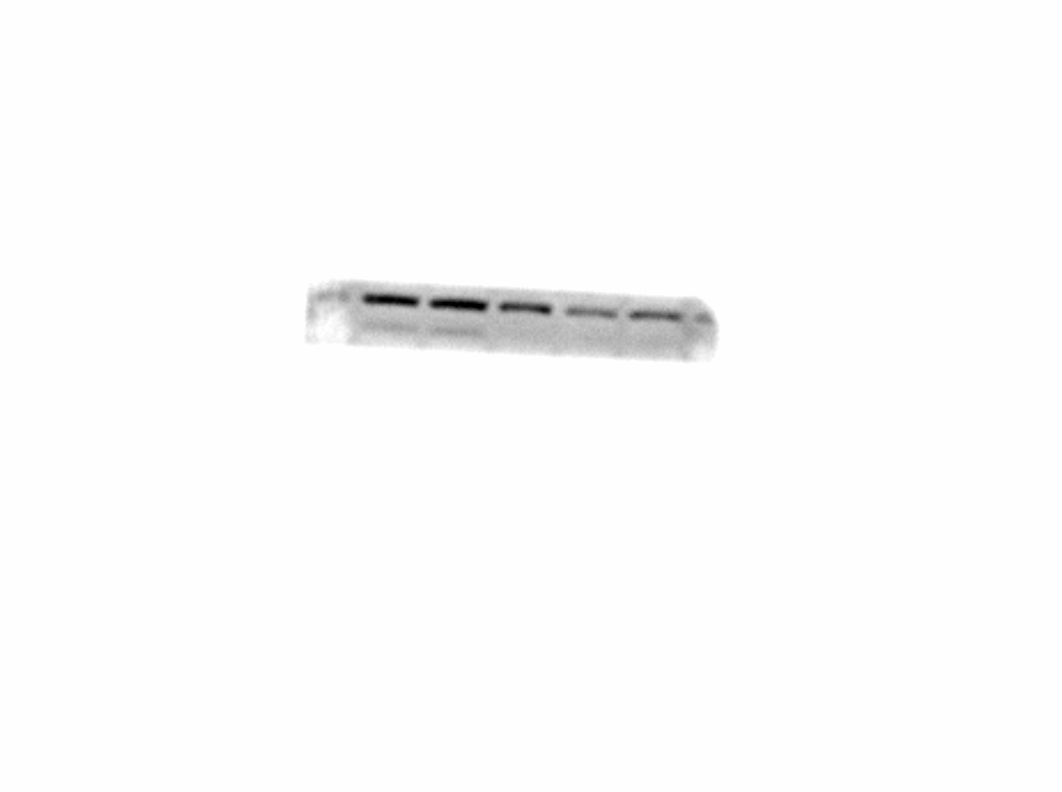

Supplement: Supplementary file 26 — original western blots-Fig4A LNCaP-pAKT [file 41420_2022_1069_MOESM26_ESM.tif]

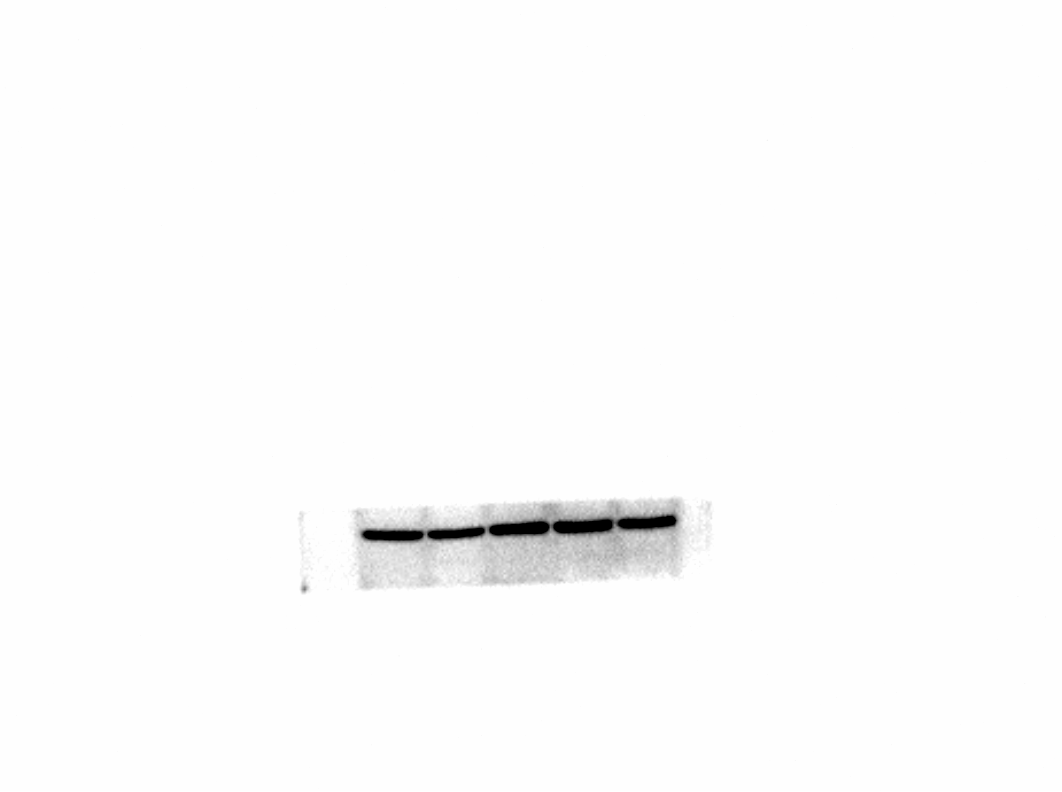

Supplement: Supplementary file 27 — original western blots-Fig4A LNCaP-beta-actin [file 41420_2022_1069_MOESM27_ESM.tif]

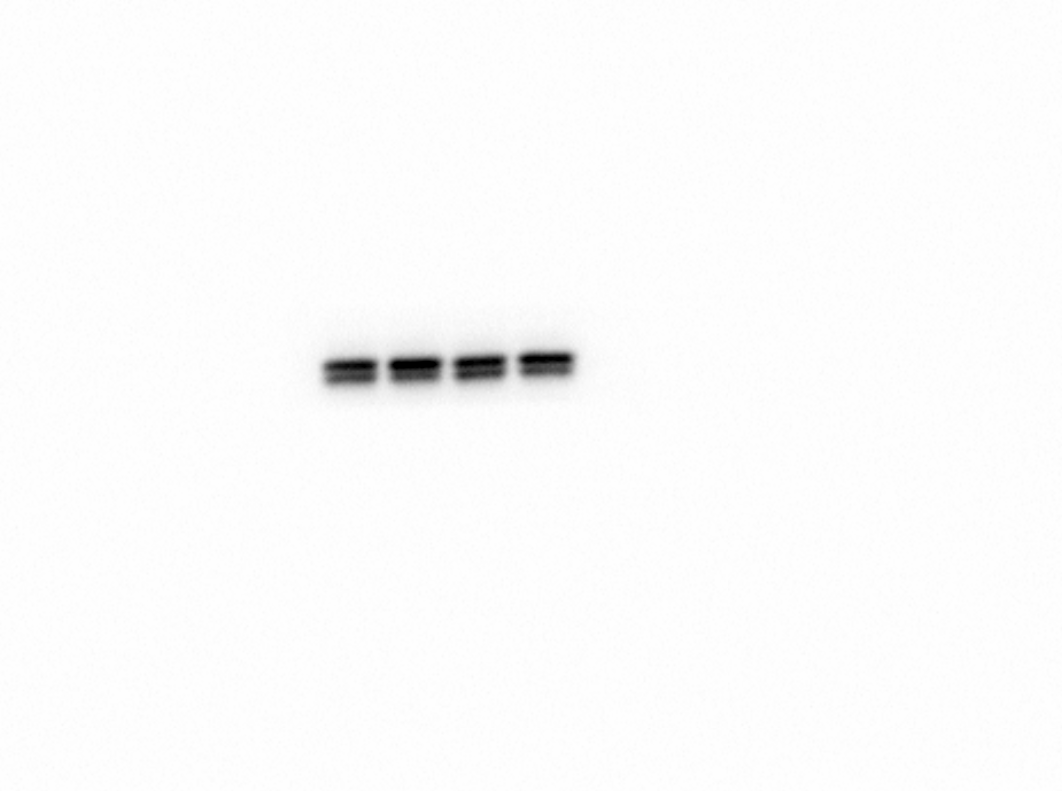

Supplement: Supplementary file 28 — original western blots-Fig4B LNCaP-AKT [file 41420_2022_1069_MOESM28_ESM.tif]

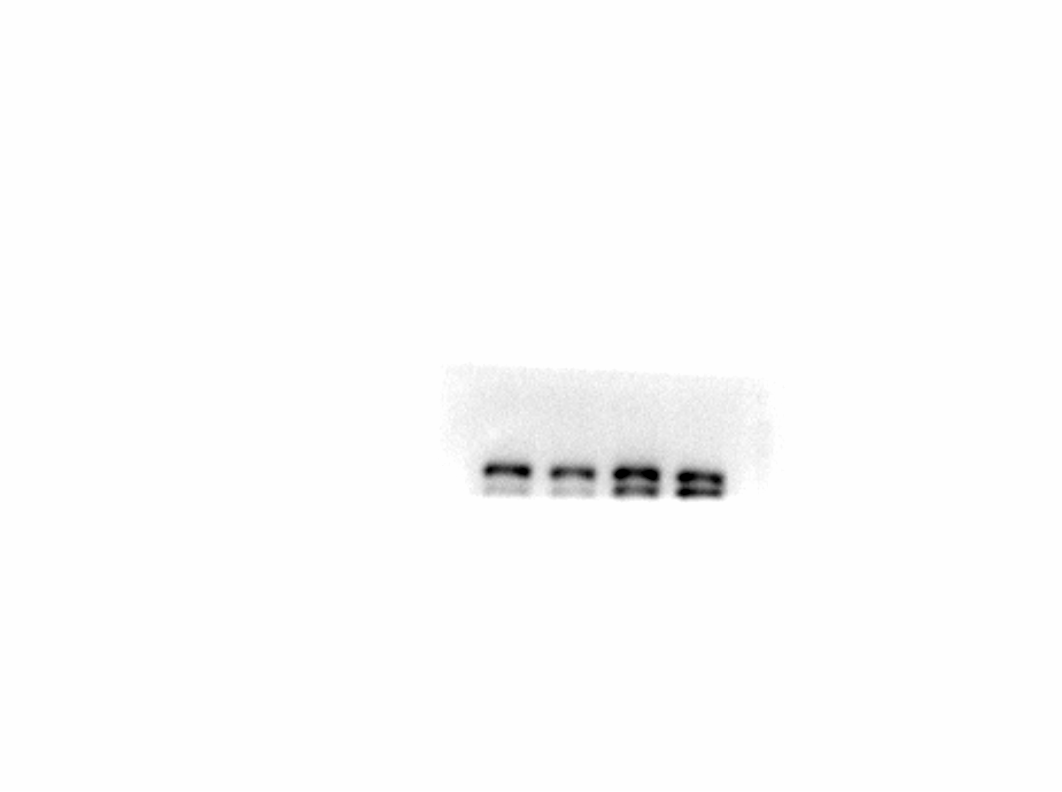

Supplement: Supplementary file 29 — original western blots-Fig4B LNCaP-RASAL2 [file 41420_2022_1069_MOESM29_ESM.tif]

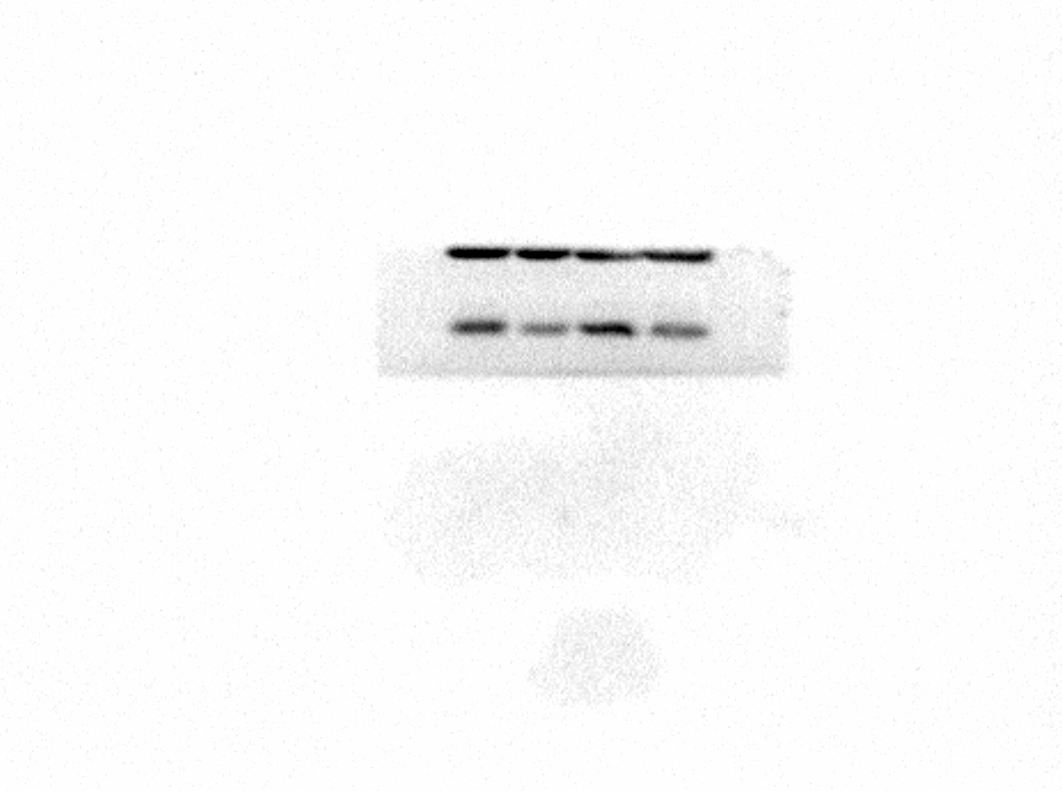

Supplement: Supplementary file 30 — original western blots-Fig4B LNCaP-cyclin D1 [file 41420_2022_1069_MOESM30_ESM.tif]

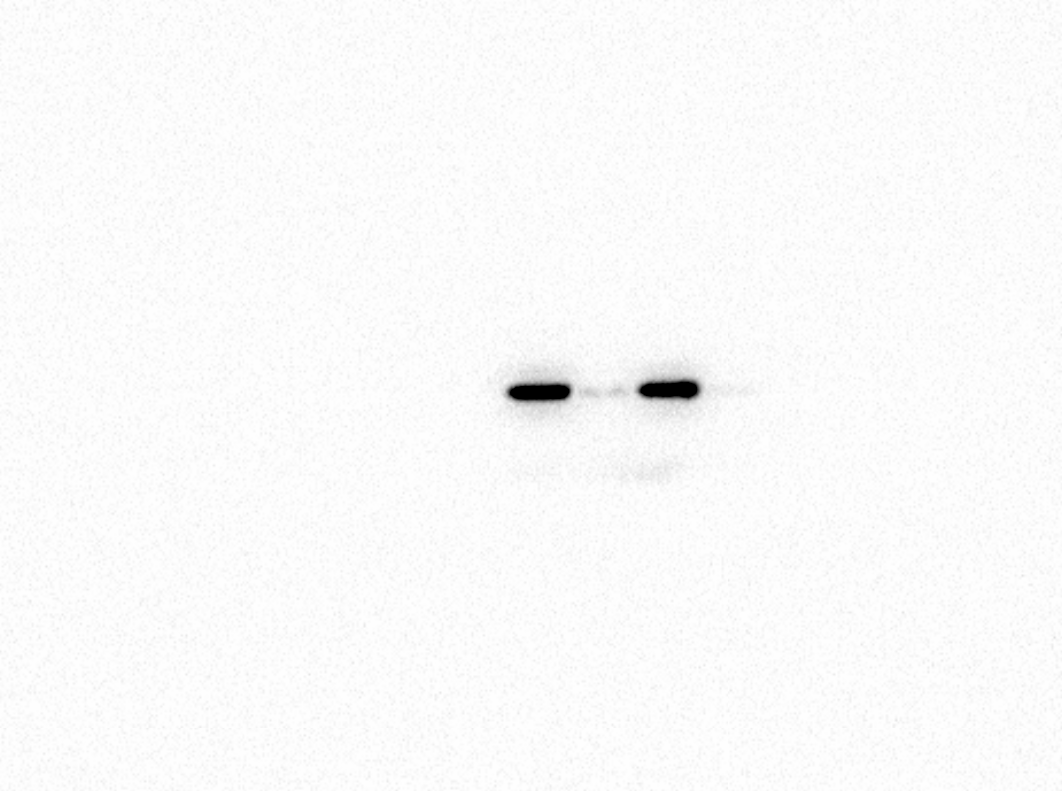

Supplement: Supplementary file 31 — original western blots-Fig4B LNCaP-pAKT [file 41420_2022_1069_MOESM31_ESM.tif]

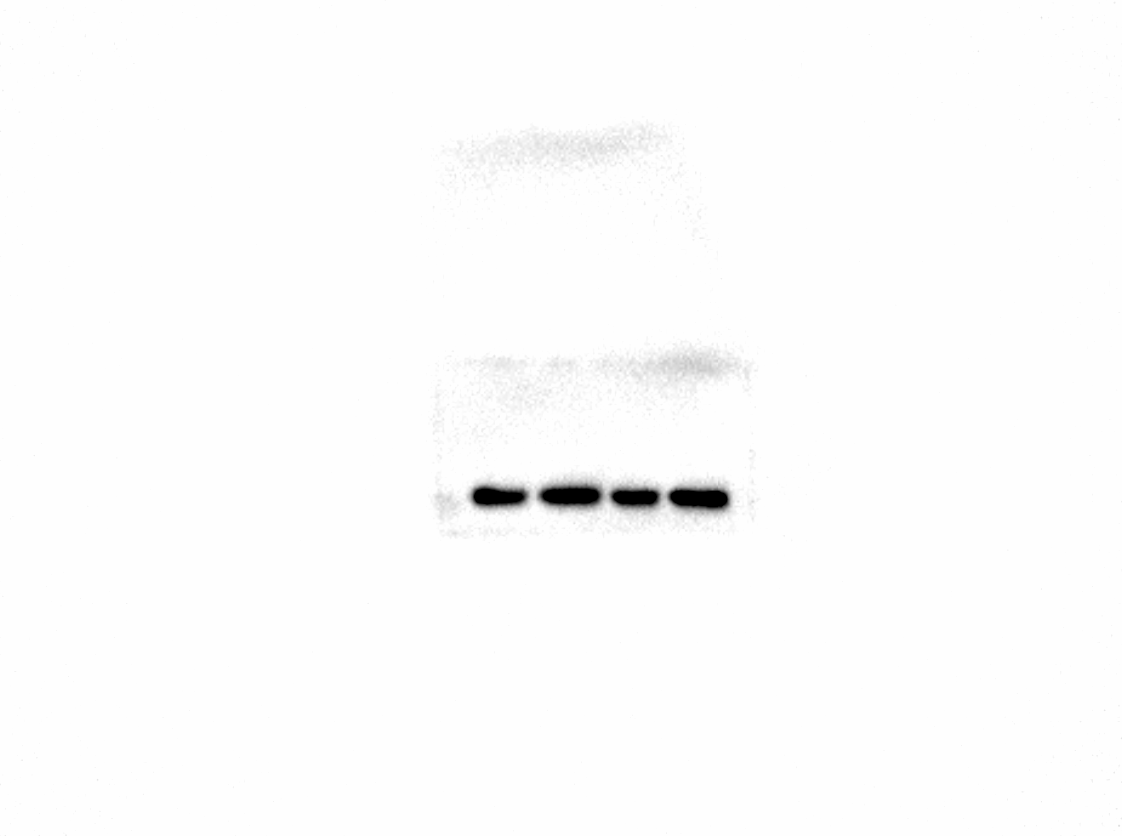

Supplement: Supplementary file 32 — original western blots-Fig4B LNCaP-beta-actin [file 41420_2022_1069_MOESM32_ESM.tif]

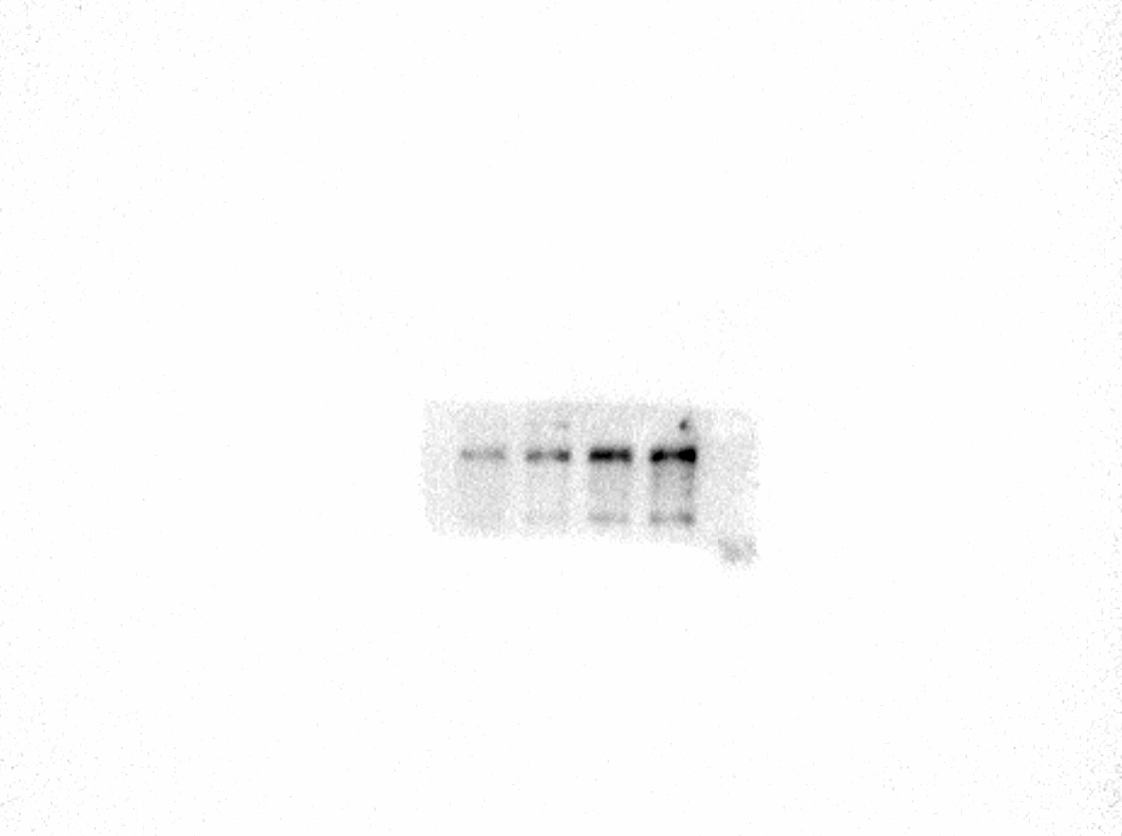

Supplement: Supplementary file 33 — original western blots-Fig4D-RASAL2 [file 41420_2022_1069_MOESM33_ESM.tif]

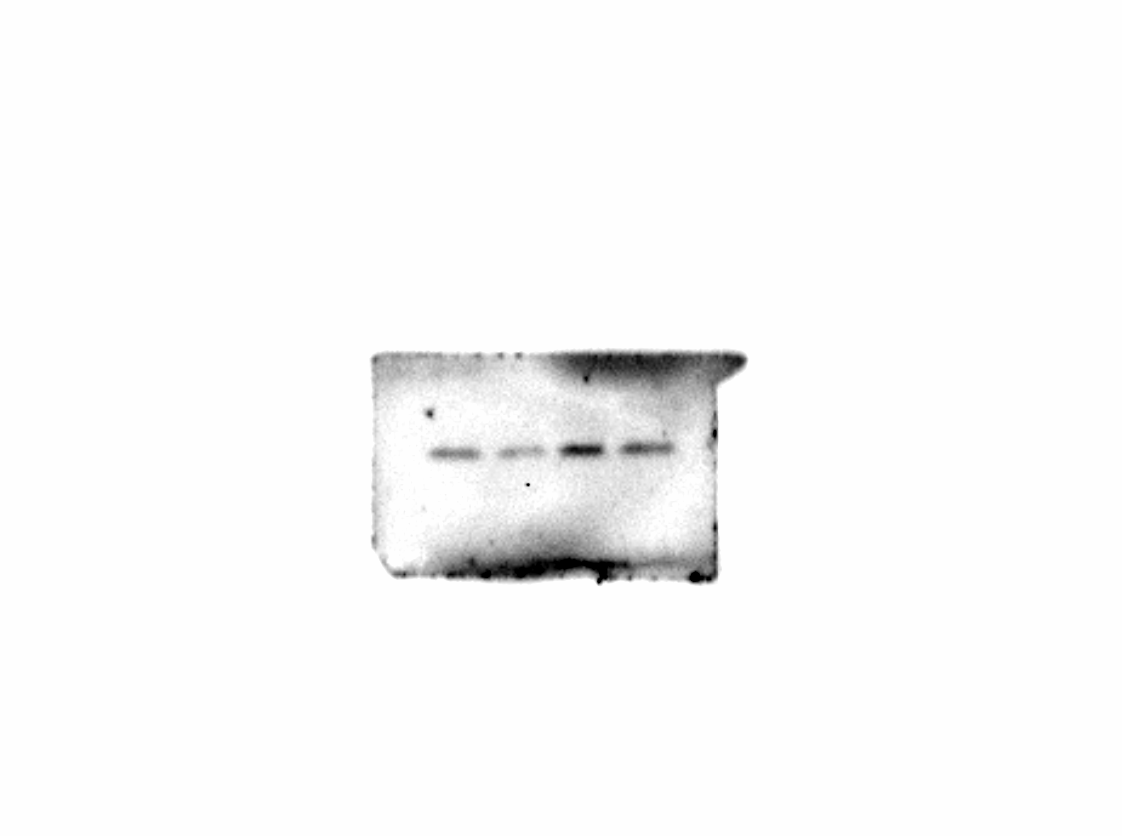

Supplement: Supplementary file 34 — original western blots-Fig4D-cyclin D1 [file 41420_2022_1069_MOESM34_ESM.tif]

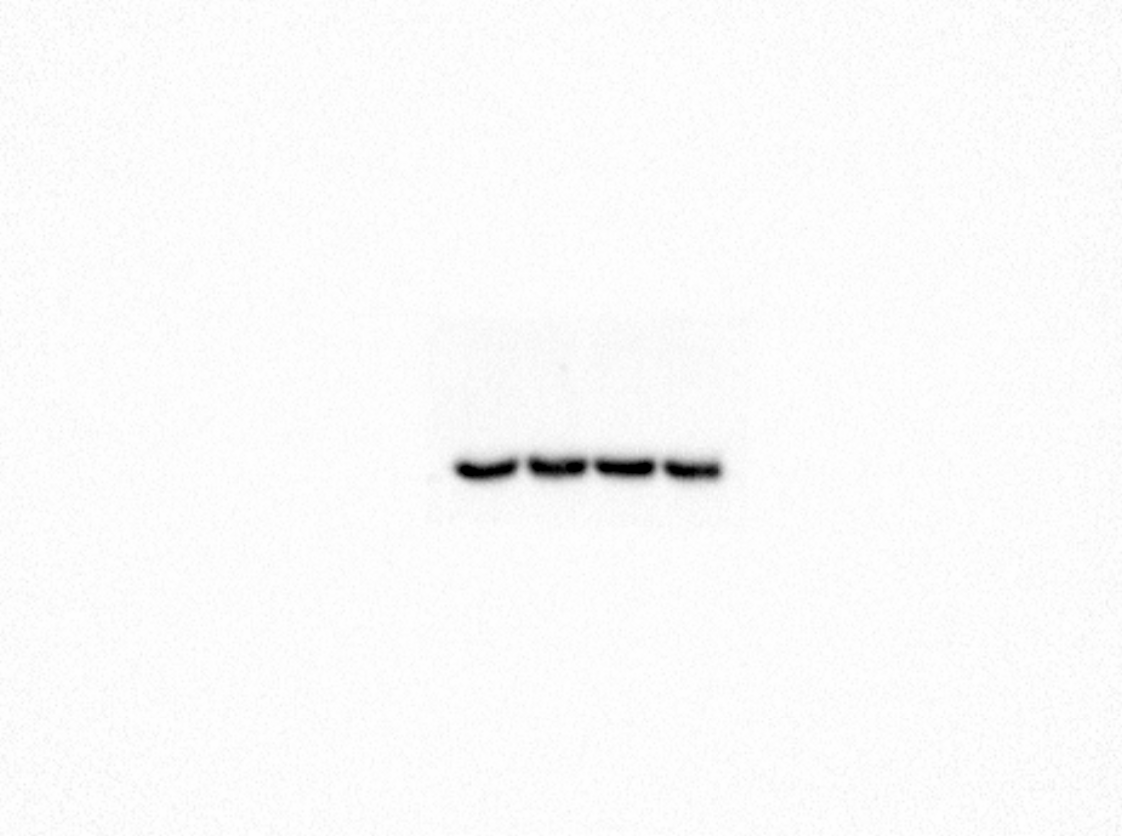

Supplement: Supplementary file 35 — original western blots-Fig4D-beta-actin [file 41420_2022_1069_MOESM35_ESM.tif]
